# Whole‐Genome Resequencing Reveals Polygenic Signatures of Directional and Balancing Selection on Alternative Migratory Life Histories

**DOI:** 10.1111/mec.17538

**Published:** 2024-11-04

**Authors:** Peter A. Moran, Thomas J. Colgan, Karl P. Phillips, Jamie Coughlan, Philip McGinnity, Thomas E. Reed

**Affiliations:** ^1^ School of Biological, Earth and Environmental Sciences University College Cork Cork Ireland; ^2^ Environmental Research Institute University College Cork Cork Ireland; ^3^ A‐LIFE, Section Ecology & Evolution Vrije Universiteit Amsterdam Amsterdam The Netherlands; ^4^ Institute of Organismic and Molecular Evolution, Johannes Gutenberg, University Mainz Mainz Germany; ^5^ Canadian Rivers Institute, University of New Brunswick Fredericton New Brunswick Canada; ^6^ Marine Institute, Furnace, Newport Mayo Ireland

**Keywords:** genome scans, local adaptation, migration, population genomics, *Salmo trutta*

## Abstract

Migration in animals and associated adaptations to contrasting environments are underpinned by complex genetic architecture. Here, we explore the genomic basis of facultative anadromy in brown trout (*Salmo trutta*), wherein some individuals migrate to sea while others remain resident in natal rivers, to better understand how alternative migratory tactics (AMTs) are maintained evolutionarily. To identify genomic variants associated with AMTs, we sequenced whole genomes for 194 individual trout from five anadromous–resident population pairs, situated above and below waterfalls, in five different Irish rivers. These waterfalls act as natural barriers to upstream migration and hence we predicted that loci underpinning AMTs should be under similar divergent selection across these replicate pairs. A sliding windows based analysis revealed a highly polygenic adaptive divergence between anadromous and resident populations, encompassing 329 differentiated genomic regions. These regions were associated with 292 genes involved in various processes crucial for AMTs, including energy homeostasis, reproduction, osmoregulation, immunity, circadian rhythm and neural function. Furthermore, examining patterns of diversity we were able to link specific genes and biological processes to putative AMT trait classes: migratory‐propensity, migratory‐lifestyle and residency. Importantly, AMT outlier regions possessed higher genetic diversity than the background genome, particularly in the anadromous group, suggesting balancing selection may play a role in maintaining genetic variation. Overall, the results from this study provide important insights into the genetic architecture of migration and the evolutionary mechanisms shaping genomic diversity within and across populations.

## 1 Introduction

Migration allows organisms to exploit spatiotemporal variation in resources and escape seasonally deteriorating environmental conditions. While migration is widespread across the animal kingdom, many species exhibit partial migration, wherein some members of a given population adopt a migratory tactic while others adopt a non‐migratory, or resident, tactic (Chapman et al. 2011). The evolution and persistence of such alternative migratory tactics (AMTs) has long fascinated evolutionary biologists, given the importance of facultative migration for life‐history adaptation and eco‐evolutionary dynamics (Dingle 2006; Gross, Coleman, and McDowall 1988; Liedvogel, Åkesson, and Bensch 2011). Migratory species may also be particularly susceptible to environmental change (Shaw 2016), so understanding how migratory traits evolve in response to anthropogenic stressors, such as artificial barriers (Zarri et al. 2022), novel pathogens/parasites (Kane et al. 2022), climate change (Pulido and Berthold 2010) and harvesting (Thériault et al. 2008) is becoming increasingly important from a conservation and management perspective.

Taxonomic groups with diverse migratory types, like salmonid fishes, offer valuable insights into the causes of AMTs (Ferguson et al. 2019). Salmonid migrations span various distances, from short freshwater movements (potamodromy) to extensive journeys from freshwater to the sea (anadromy). Migratory propensity can itself vary across species, populations, individuals (e.g., sex and body size) and over time within populations (Dodson et al. 2013; Ferguson et al. 2019; Lavender et al. 2023; Sloat et al. 2014), impacting population structure and potential for local adaptation (Quéméré et al. 2016; Rougemont et al. 2023). Phylogenetic evidence suggests that anadromy evolved at least twice from freshwater salmonid ancestors (Alexandrou et al. 2013). Although many migratory‐related traits have been found to be heritable (Debes et al. 2020; Hecht et al. 2015; Reed et al. 2019; Thériault et al. 2007), elucidating the genetic basis of AMTs has been challenging. The shift between migratory forms is a conditional strategy that occurs when specific threshold values of status traits (e.g., physiological condition) are reached (Phillis et al. 2016; Roff 1996), which means that a large fraction of overall phenotypic variation will be explained by environmental, rather than genetic, effects. Accurately determining AMT phenotypes (i.e., whether individuals are migratory versus resident) can itself often be difficult, as individuals sampled early in life (e.g., during freshwater rearing) might appear outwardly undifferentiated yet be on distinct developmental trajectories. Moreover, the ‘migration syndrome’ involves a complex interplay of developmental, physiological and behavioural processes (Dingle 2006) and is, thus, expected to be highly polygenic.

Previous studies investigating the genetic basis of migration‐related or life‐history traits in salmonids have revealed diverse genetic architectures. Studies focussed on specific well‐defined phenotypes, such as age at maturity, migration distance and timing of migration or spawning, have generally identified genes or regions with large effects (Ayllon et al. 2015; Barson et al. 2015; Hecht et al. 2012; Lemopoulos et al. 2019; Micheletti et al. 2018; Prince et al. 2017; Thompson et al. 2019). In contrast, studies broadly comparing migratory and resident ecotypes (Ferchaud et al. 2014; Hale et al. 2013; Hecht et al. 2013; Kjærner‐Semb et al. 2020; Lemopoulos et al. 2018; Perrier et al. 2013; Salisbury et al. 2022; Tigano and Russello 2022; Veale and Russello 2017) have often pointed towards more polygenic architecture (but see Arostegui et al. 2019; Pearse et al. 2019). These contrasting patterns might, in part, be explained by the fact that a whole suite of interrelated traits is under divergent selection between migratory and resident populations, including migration propensity itself but also traits governing migratory performance and life at sea, such as navigational abilities, energy homeostasis, smoltification, osmoregulatory capacity, thermal tolerance, omega‐3 metabolism, marine growth rate and resistance to marine pathogens/parasites.

In this study, we focus on brown trout (*Salmo trutta*), an iconic species of high economic and conservation importance, in which AMTs and associated traits are highly variable within and between populations (Klemetsen et al. 2003; Nevoux et al. 2019; Ferguson et al. 2019). To identify genomic regions associated with AMTs, we targeted multiple pairs of landlocked resident populations and corresponding anadromous populations with unfettered access to the sea. Specifically, we focused on trout populations separated by natural waterfalls in five different rivers along the west coast of Ireland (~ 300 km range). Following the last glacial recession, several anadromous brown trout lineages recolonised these catchments (Ferguson 2007; McKeown et al. 2010) and in some places, as the land rose due to isostatic rebound, populations became isolated above natural falls (Shennan, Bradley, and Edwards 2018). As migratory individuals may leave but never return (owing to the falls being impassable in the upstream direction), genetic variants associated with the propensity to migrate are expected to have been selected against in these resident populations, leading to the erosion or loss of anadromy and associated adaptations to marine life.

Following a similar study design and logic to previous population genomic studies (Perrier et al. 2013; Kjærner‐Semb et al. 2020; Pearse et al. 2019; Arostegui et al. 2019; Clare et al. 2023; Veale and Russello 2017; Tigano and Russello 2022), we hypothesised that positive selection should favour alleles conferring increased migratory propensity in our anadromous (below‐falls) populations of *S. trutta*, while strong negative selection against such alleles should occur in the resident (above‐falls) populations. Allele frequencies at such migration‐propensity loci should thus be divergent between anadromous and resident populations and signatures of parallel evolution at the nucleotide or gene level may also be expected (Arostegui et al. 2019; Pearse et al. 2014; Taylor, Foote, and Wood 1996). We, therefore, anticipated reduced diversity in genomic regions associated with migration propensity in both anadromous and resident populations, as selective sweeps for alternative alleles may have occurred in each ecological context leaving a signature of reduced polymorphism at linked sites (Nielsen 2005). Alternatively, balancing selection may arise for some genomic regions associated with AMTs in the below‐falls contexts if a mix of migratory and resident types can coexist. For example, sexual conflict between AMTs might occur, because alleles conferring increased migratory propensity might be positively selected in females but negatively selected in males (Fleming and Reynolds 2004). In this case, balancing selection on migration‐propensity loci in anadromous populations might elevate diversity in these genomic regions relative to resident populations, where residents are presumably consistently favoured (Table 2). In contrast, genes associated with migratory lifestyle or adaptation to saltwater habitats should experience relaxed selection in resident populations; thus, we expect a pattern of elevated diversity (due to mutation accumulation under drift) relative to the same genomic regions in the anadromous populations.

Our overall aims were, thus, to (1) investigate the genetic architecture of AMTs using genome scans based on low coverage whole‐genome (re)sequencing (lcWGR); (2) determine if genes found in regions under selection are enriched for specific biological processes, identify the genes associated with outlier genomic regions, and assess their overlap with previously identified candidate AMT genes and (3) compare patterns of diversity across the genome between anadromous and resident populations, in order to understand the types of selection (directional, balancing and relaxed) acting on genomic regions putatively associated with migration propensity or migration lifestyle.

## 2 Methods

### 2.1 Sample Collection

**FIGURE 1:**
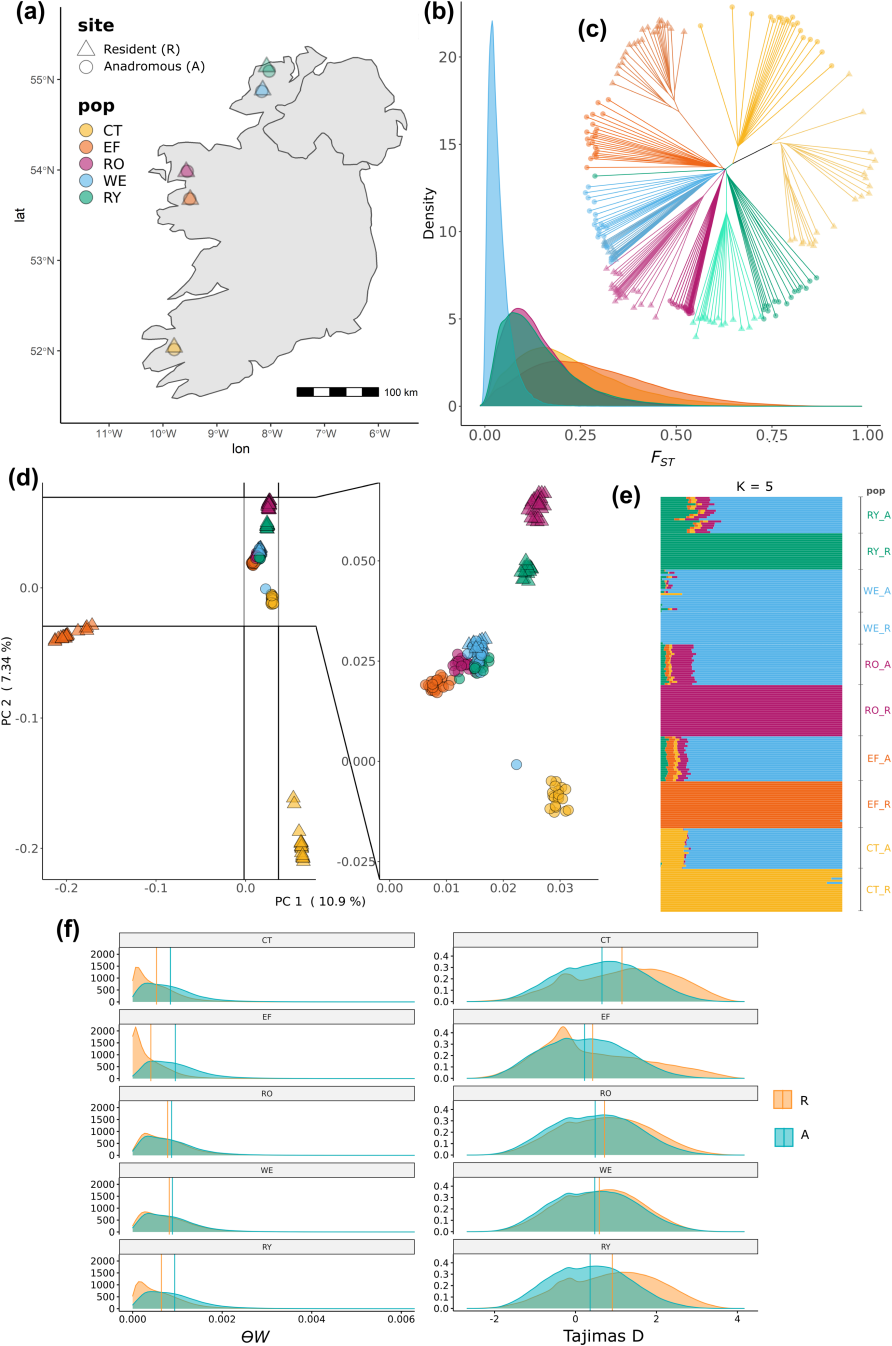
Overview of population genetic structure. (a) Map of the 10 paired sampling locations, including sites above (triangles) and below (circles) natural waterfalls, along the west coast of Ireland. Colours correspond to the sampling locations provided in the legend, which corresponds to the river systems outlined in Table 1. (b) Genetic differentiation (*F*_*ST*_) between fish sampled above and below falls, with *F*_*ST*_ estimated in 10 kb non‐overlapping sliding windows across the genome. (c) Neighbour‐joining tree based on pairwise genetic distances. Note one individual sampled from RY_B was a sea trout that may have migrated from another population as it clusters separately. (d) Principal component analysis (PCA) showing the distribution of genomic variation on PC1 and PC2. (e) Individual admixture proportions for K = 5 (admixture plots for K = 2–10 provided in Figure S6). (f) The distribution of genetic diversity (ϴW) and Tajima's D, calculated in 10 kb non‐overlapping windows, among fish sampled above and below falls within each river. For all population comparisons, the above falls had lower genetic diversity and higher Tajima's D.

Fish were sampled using electrofishing from five rivers along the west coast of Ireland during the summer of 2018 (Figure 1A; Table 1). For each river, samples were taken from two sites, one above and the other below natural waterfalls, which are expected to act as impassable barriers to upstream migration. Thus, fish sampled above waterfalls are expected to have experienced strong historical (and possibly ongoing) selection against migration. Given that migratory phenotypes and associated genotypes are expected to be purged from this population leading to the establishment of resident populations above the falls, we refer to these as ‘resident’ populations. In contrast, fish sampled below falls are predicted to exhibit a higher frequency of genotypes associated with seaward migration, and, therefore, we refer to these populations as ‘anadromous’. All fish were sampled at the same developmental stage (parr), except for the Erriff River below the falls (EF B), which consisted entirely of smolts, and two adult sea trout collected below the falls in the Ray (RY B) and Gweebarra (WE B) rivers, respectively. From each sampled fish, a fin clip was taken and stored in 100% ethanol in a −80°C freezer prior to DNA extractions.

**TABLE 1:** Summary of sample information for trout used in the present population genomic‐based analysis, including sampling locations (river, site (A, anadromous; R, resident), population identifiers (Pop ID), geographical coordinates (longitude (Lon) and latitude (Lat)), number of samples by sex (N), nucleotide diversity (π × 10^−3^), Watterson's theta (ϴW × 10^−3^) and Tajima's D).

River	Site	Pop ID	Lon	Lat	*N* (female, male)	π	ϴW	Tajima's D
Cottoners	A	CT_A	−9.79574	52.04034	19 (9, 10)	1.068	0.840	0.664
	R	CT_R	−9.79086	52.01595	20 (10, 10)	0.784	0.531	1.167
Erriff	A	EF_A	−9.49597	53.67508	21 (12, 9)	1.092	0.949	0.228
	R	EF_R	−9.50474	53.6813	22 (12, 10)	0.515	0.403	0.429
Rough	A	RO_A	−9.5691	53.98173	19 (6, 13)	1.061	0.864	0.492
	R	RO_R	−9.55034	53.98546	24 (11, 13)	1.020	0.781	0.731
Ray	A	RY_A	−8.07558	55.14119	17 (10, 7)	1.103	0.935	0.368
	R	RY_R	−8.02834	55.09294	17 (8, 9)	0.874	0.639	0.923
Gweebarra	A	WE_A	−8.15162	54.88512	20 (10, 10)	1.079	0.883	0.485
	R	WE_R	−8.16692	54.8693	15 (8, 7)	1.018	0.816	0.602

### 2.2 DNA Extraction, Library Preparation and Sequencing

A preliminary genetic analysis using microsatellites was performed to identify and remove highly related individuals and to genetically sex individuals to ensure an equal sex ratio per population for sequencing (sex marker: Prodöhl et al. unpublished). Genomic DNA was extracted from fin clips using a Qiagen DNeasy Blood & Tissue Kit (Qiagen, Hilden, Germany). Extracted DNA was assessed for quality and concentration using a Nanodrop ND‐1000 spectrometer followed by a Qubit fluorometer using a dsDNA Broad Range (BR) assay kit (Thermo Scientific). Samples were shipped to a commercial sequencing company (Novogene, UK) for further quality assessment before individual PCR‐free genomic libraries were prepared using a NEB Next Ultra II DNA library preparation kit. All libraries were individually indexed, multiplexed and sequenced (paired‐end sequencing: 2 × 150 bp) on an Illumina Novaseq6000. For each sample, sequencing resulted in the generation of approximately 46.4 million PE reads (predicted coverage 5.9X; Table S1). All whole‐genome resequencing data are available from the European Nucleotide Archive (ENA) (BioProject ID: PRJEB72781).

### 2.3 Quality Assessment, Sequence Filtering and Alignment

The base quality of sequenced data was first examined using FastQC (v0.11.5, Andrews 2010) and reports visualised using MultiQC (v1.7, Ewels et al. 2016) to determine sequences of low quality and presence of adaptor contamination. Low‐quality bases and reads were filtered using fastp (v0.20.0, Chen et al. 2018) with default parameters aside from allowing a minimum base quality of 20 (‐q 20), an ‘n’ (ambiguous) base limit of 15 (‐n 15), minimum read length of 50 (‐l 50), enabled overrepresented sequence analysis (‐p) and automatic‐adapter detection for PE data (‐‐detect_adapter_for_pe). The quality and impact of read filtering was examined for all samples by running FastQC again with the results visualised using MultiQC, confirming that filtering was sufficient and, thereby, ensuring high‐quality data for alignment. For each sample, filtered reads were mapped to the latest available brown trout reference genome assembly (NCBI assembly accession: GCF_901001165.1; Hansen et al. 2021) using Bowtie2 (v2.3.5.1, Langmead and Salzberg 2012) with default parameters aside from specifying the performance of local read alignment and setting a maximum fragment length of 700 (‐X 700). The quality of alignments and overall mapping statistics were examined using Qualimap2 (Okonechnikov and García‐Alcalde 2016). The mean number of PE reads mapped per sample was 44.7 million (mean percentage (%) of reads aligned: 99%) and the mean genome‐wide coverage was 4.99X (Table S2; Figure S1). The resulting alignment files in SAM format were sorted and converted to BAM files using SAMtools (v1.9, Li et al. 2009). The BAM files were then analysed using ANGSD (v0.912, Korneliussen, Albrechtsen, and Nielsen 2014). As an initial step, we filtered each BAM file to remove low‐quality reads and bases (ANGSD parameters: remove_bads 1, ‐trim 0, ‐minMapQ 20 and ‐minQ 20), reads that did not map uniquely (‐uniqueOnly 1) and read pairs where both could not be properly mapped (‐only_proper_pairs 1), following adjustments for the effect of excessive mismatches (‐C 50) and indels (‐baq 1). Only sites with a minimum global read depth across all samples equal to the total number of samples (*n* = 194) (thus, equivalent to ~ 1× coverage per sample) and up to a maximum global depth of *n* * 10 were retained.

### 2.4 Population Genomic Analyses

#### 2.4.1 Population Structure

To account for the genotypic uncertainty associated with low coverage sequencing, we based our analyses on genotype likelihoods (unless otherwise stated) estimated by ANGSD. To determine overall population structure, we used a series of population genomic approaches using modified scripts generated by Moran et al. (2024). First, we performed a global genome‐wide SNP calling using all 194 samples (‐SNP_pval 1e−6), applying the same set of quality filters as outlined above, with bases filtered if they were absent from > 50% of individuals (‐minInd 0.5), resulting in 10,243,067 SNPs. Genotype likelihoods for these SNPs were then calculated using the SAMtools model in ANGSD (parameters: ‐GL 1) under the assumption of Hardy–Weinberg equilibrium. Next, we performed a principal component analysis on the covariance matrix of individual allele frequencies using PCAngsd (v0.981, Meisner and Albrechtsen 2018). As a complementary measure, we used a clustering‐based approach to calculate individual admixture proportions using NGSadmix (Skotte, Korneliussen, and Albrechtsen 2013). To estimate the best predicted number of subpopulations (*K*), we ran NGSadmix for *K =* 2–10 with 10 replicates per run and then examined the log‐likelihood values using both the ΔK method (Evanno, Regnaut, and Goudet 2005) and the mean posterior probability likelihood (ln(Pr(X|K))) (Pritchard, Stephens, and Donnelly 2000). To test the role of geographic distance in contributing to population genetic structure, we calculated genetic distances using ngsDist in ANGSD (based on genotype probabilities, parameters: ‐doGeno 8, ‐doPost 1; Vieira et al. 2016) and implemented Mantel tests using the R package vegan (v.2.6.4, Oksanen et al. 2020). Lastly, to visualise population genetic structure, we created a neighbour‐joining tree based on pairwise genetic distances using FastME (v. 2.0, Lefort, Desper, and Gascuel 2015) and ggtree (v.1, Yu et al. 2017).

To compare anadromous and resident groups, we first performed global (pooled populations by ecotype: anadromous vs. resident) *F*_*ST*_ comparisons, followed by a local (river‐specific) approach to examine genomic diversity and differentiation. For both global and local comparisons, we first calculated Sample Allele Frequency likelihoods (SAF) for each location. At the global level, each location represents an ecotype, while at the local level, each location represents a population. We used the same quality filters as previously described, but excluded SNP calling to avoid biasing the site frequency spectrum (SFS) and applied a stricter threshold for missing data (parameters: ‐minInd 0.8, ‐doSaf 1, ‐doMajorMinor 4) (Korneliussen, Albrechtsen, and Nielsen 2014; Lou et al. 2021). Second, using these SAF values, we computed the folded SFS with the reference genome assembly as the ancestral state using realSFS (Fumagalli 2013). The genome‐wide SFS served as a prior for calculating differentiation (*F*_*ST*_) and diversity statistics, including Watterson's theta (ϴW), pairwise diversity (π) and Tajima's D, in non‐overlapping 10 kb sliding windows across the genome. For local diversity statistics, we computed a separate SFS for each of the 10 populations. For global *F*_*ST*_ estimates, we used the joint SFS (2D‐SFS) to compare the anadromous and resident ecotypes. For local *F*_*ST*_ estimates, we computed the 2D‐SFS for the corresponding populations within each river.

#### 2.4.2 Genomic Scans for Signatures of Selection

To identify genomic regions with a high probability of adaptive divergence between anadromous and resident ecotypes, we employed two genome‐based scan approaches on 194 individuals from five populations per ecotype (96 anadromous and 98 resident individuals; Figure S2). First, we used a global approach to identify regions of the genome that showed consistent differentiation between anadromous and resident populations. We calculated *F*_*ST*_ in 10 kb non‐overlapping windows for the anadromous and resident pools using realSFS in ANGSD (v0.94). Windows with fewer than 500 (variant or invariant) sites were excluded to reduce the potential impact of regions with low amounts of data. Outlier windows were identified as those falling within the top 1% quantile (*F*_*ST*_ > 0.16, *n* = 1385 outlier windows). Second, as pooling individuals from different populations could introduce biases in outlier detection due to the Wahlund effect, we employed a Bayesian approach implemented in BayPass v2.2 (Gautier 2015). Specifically, we utilised the contrast statistic (C2), a powerful method for identifying significantly differentiated loci between populations, while mitigating false positives by controlling for demographic history (Olazcuaga et al. 2021). To generate the input for Baypass, we first performed a global SNP call to obtain a set of high‐quality SNPs across all populations (ANGSD parameters: ‐MAF 0.05, ‐SNP_pval 1e−6, ‐minind 0.8, ‐minQ 30 and ‐minMapQ 30, ‐domajorminor 1), resulting in the retention of 1,467,659 SNPs. Using these sites, we calculated the minor allele frequency (MAF) based on genotype likelihoods for each population (‐domajorminor 3) and then combined the outputs from all populations into the Baypass format, utilising modified scripts from Mérot et al. (2021). We ran the standard contrast model (C2 model) in Baypass three times with different seeds to ensure robustness of the results. To establish a significance threshold, we simulated 10,000 Pseudo Observed Data (PODs) using the simulate.baypass() function in the baypass_utils.R script provided in the BayPass package. Subsequently, we re‐ran BayPass (C2 model) using the C2 PODs, with the top 1% quantile used as the significance threshold.

To identify high‐confidence outlier regions associated with AMTs, we focused on loci identified as outliers by both genome‐based scan approaches (*F*_*ST*_ and BayPass C2) and refer to these as AMT outliers. Genes within or near (10 kb) these regions were labelled as AMT‐associated genes. This dual approach aimed to ensure a stringent selection of candidate loci, minimising the influence of potential confounding factors. Additionally, we conducted per‐river (local) *F*_*ST*_ analyses and found both approaches gave broadly similar results (see details in Appendix S1; Figures S10 and S11), which suggests minimal impact from population structure or the Wahlund effect.

#### 2.4.3 Functional Impact

To assess the functional role of putative AMT‐associated genes, we obtained Gene Ontology (GO) terms using biomaRt (v.2.57.1, Durinck et al. 2009) and performed GO term enrichment analyses using TopGo (v. 2.54.0, Alexa and Rahnenfuhrer 2023). Using biomaRt, we obtained GO term annotations from the zebrafish (*Danio rerio*) from Ensembl and assigned GO terms to homologous genes in brown trout, as the zebrafish has a better annotated reference genome assembly compared to *S. trutta*. To perform the enrichment analysis, we tested for all three ontology types (biological processes (BP), molecular function (MF) and cellular component (CC)), using Fisher's exact test with a node size of 10 and the ‘weight01’ algorithm. The significantly enriched GO terms (*p* < 0.01) were visualised by creating network plots in Cytoscape V3.10.1 (Shannon et al. 2003).

To understand the potential functional impact of specific polymorphic sites that may be under selection, SNPs with signatures of selection were functionally annotated using snpEff (v. 4.2) (Cingolani 2022). To improve our ability to link outlier genes with AMTs and to reduce putative false‐positives, we tested whether any of our AMT‐associated genes overlapped with a set of candidate genes that were previously found to be differentially expressed between brown trout smolts versus non‐smolts reared in a common garden environment (Wynne et al. 2021), which originated from two of the populations included in the present analysis. In addition, we further annotated our candidate genes using the ZFIN and UniProt databases and performed literature searches to determine if any of our outlier genes overlapped with candidate genes from previous studies on anadromy in salmonids.

#### 2.4.4 Genetic Diversity Patterns and the Nature of Divergent Selection on AMT Outliers

##### 2.4.4.1 Using Tajima's D to Categorise AMT Outliers Into Putative Trait Classes

To understand the types of selection acting on putative AMT‐associated regions, we estimated Tajima's D (*TD*) in non‐overlapping 10 kb windows using ANGSD for each of the 10 populations. To reduce noise, windows with fewer than 500 sites (variant and invariant) were excluded (average number of sites per 10 kb window = 7164). Following Kjærner‐Semb et al. (2020), we standardised *TD* to have a mean of zero and standard deviation of one (Z‐scores), allowing windows with *ZTD* > 0 to be considered above the genome‐wide average for that population and windows with *ZTD* < 0 below the genome‐wide average. *ZTD* scores were then directly comparable across populations, mitigating against confounding factors such as population differences in demographic history and genetic diversity.

Average *ZTD* scores were then calculated for each AMT outlier window (*n* = 329) separately for the resident and the anadromous pools. Windows with average *ZTD* values in the lower tertile of the respective *ZTD* distributions were classified as ‘low’, while windows in the middle and upper tertiles were classified as ‘mid‐to‐high’. We then split the AMT outliers into four groups (Table 2). (i) We reasoned that AMT windows experiencing opposing directional selection in anadromous versus resident populations (e.g., migration propensity loci) should exhibit low *ZTD* in the anadromous pool and low *ZTD* in the resident pool (*LA_LR*) because both positive and negative selection are expected to lead to an excess of low‐frequency polymorphisms (and hence low *ZTD*) relative to the population average. (ii) A second set of genes might be expressed only in migratory individuals (e.g., migratory lifestyle or saltwater adaptation loci), and thus should experience directional selection in anadromous populations and relaxed selection in resident populations. Thus, we expect AMT outliers belonging to this category to exhibit low *ZTD* in the anadromous pool and mid‐to‐high *ZTD* in the resident pool (*LA_MHR*). (iii) A third class of genes, related to residency, might have enhanced expression in residents or remain inactive in migrants (e.g., loci influencing early maturation). Consequently, AMT outliers in this category should undergo directional selection in resident populations and relaxed selection in anadromous populations, placing them in the mid‐to‐high *ZTD* in the anadromous pool and low *ZTD* in the resident pool (*MHA_LR*) grouping. (iv) Finally, AMT outliers not falling into any of the previous three categories must fall into the fourth category: mid‐to‐high *ZTD* in both pools (M*HA_MHR*). For example, balancing selection on some loci in this category might occur in both anadromous and resident populations, resulting in an excess of intermediate frequency variants, with the balance tipping more towards one variant in anadromous populations and another variant in resident populations (hence they show up as AMT outliers). Alternatively, balancing selection may occur in anadromous populations and relaxed selection in resident populations (or vice versa), such that *ZTD* is intermediate‐to‐high in both. In general, it is important to bear in mind that these four groupings are only loose categories meant to aid in the interpretation of heterogenous diversity patterns across AMT outliers.

**TABLE 2:** Predictions for locus types assigned to groups based on Tajima's D in relation to migration (propensity and lifestyle) and residency in anadromous and resident fish populations.

Type of allele/locus	Prediction for anadromous (A) pops			Prediction for resident (R) pops
Phenotypic composition	Residents rare	Mix of migrants and residents	Group	Migrants rare
Migration propensity loci	Directional selection	Balancing selection	LA_LR/(MHA_LR)	Directional selection
Migration lifestyle loci expressed in migrants only^a^	Directional selection	Directional selection (but more drift)	LA_MHR	Relaxed selection (drift only)
Resident loci	Drift/weak selection	Balancing selection	MHA_LR	Directional selection

^a^ Note that if migration lifestyle alleles/loci are also expressed in residents, they are likely to have negative fitness effects on residents and positive fitness effects on migrants (antagonistic pleiotropy). In this case, the predictions should be more similar to migration propensity alleles/loci (top row). Note for resident alleles/loci as all individuals will spend some of their life in freshwater some traits associated with residency are likely to be expressed and under selection in both resident and migratory individuals.

To ensure our results were not biased by misaligned reads (given the highly duplicated nature of salmonid genomes (Lien et al. 2016; Dallaire et al. 2023)), we implemented three additional analyses. First, we assessed the proportion of paralogs in outlier regions relative to the genomic background using a chi‐squared test with Yates' continuity correction. Second, we compared read depth in outlier regions against the background genome using Wilcoxon rank‐sum test. Finally, we used ngsParalog (https://github.com/tplinderoth/ngsParalog) to remove potential paralogs from the dataset and reanalysed it, comparing diversity estimates between AMT regions and the genomic background (Figure S3). Additionally, we visually compared the per‐population SFS before and after paralog filtering (Figure S4). In summary, our analysis revealed no enrichment of paralogs in outlier regions, consistent mean read depth and no discernible effect of paralog filtering on diversity estimates (details in Appendix S1). These findings collectively indicate that paralogs or duplicated loci do not exert a substantial influence on our outlier set and we present results based on the original unfiltered data here.

##### 2.4.4.2 Do Some AMT Outliers Experience Balancing Selection?

To further probe for balancing selection among AMT outliers, we used three approaches. First, we tested whether *ZTD* scores were on average higher for AMT outliers compared to the background genome (i.e., non‐AMT outlier windows) in the anadromous and resident pools separately. We expected stronger evidence for balancing selection on AMT outliers (i.e., *ZTD* being more positive compared to background genome) in the anadromous pool, because migration may be more strongly favoured in some individuals (e.g., females, individuals in poor early‐life condition) than in others, while selection should uniformly act against migrants in resident populations. Additionally, we examined *ZTD* correlations between anadromous and resident populations for AMT outliers and the background genome separately.

Second, we examined whether any of our AMT outliers are in the 99th percentile of the genome‐wide *ZTD* distribution in either the anadromous or resident pools. We ran simple chi‐squared tests on each pool to test whether the fraction of AMT outliers falling into the 99th percentile of the *ZTD* distribution was more than expected by chance.

Third, we tested whether AMT outliers are under long‐term balancing selection using the program Betascan (Siewert and Voight 2017), which summarises allele frequency correlations across windows, using a statistic known as β, with regions under balancing selection predicted to be in the 99th percentile of β scores. As input, we converted VCF files from each population to Betascan format using glactools (Renaud 2018) and calculated β scores for each population separately in 10 kb windows. To mitigate false‐positives, SNPs with very low or high frequencies (< 0.1 or > 0.9) were excluded (Siewert and Voight 2017). To compare anadromous and resident pools, we transformed β scores into Z‐scores (applying the same approach we used for *TD*) and examined whether any of our AMT outliers were in the 99th percentile of the chromosome‐wide β scores in either the anadromous or resident pools.

## 3 Results

### 3.1 Gene Flow and Genetic Drift Drive Population Structure

A PCA of 194 trout genomes sampled from replicate above and below natural waterfalls revealed clear population structuring (Figure 1D). The resident populations (above falls) were highly divergent, with EF and CT residents separated most strongly on PC1 (10.9% of the variation) and PC2 (7.34%). Further separation between the RY and RO resident populations was revealed on PC3 (5.06%) and PC4 (4.14%) (Figure S5). The greater divergence observed among resident populations likely reflects reduced gene flow, lower effective population sizes and increased drift. In contrast, there was greater clustering among the anadromous (below falls) populations, likely reflecting higher gene flow and connectivity. Genetic differentiation between above versus below falls populations within each river was high, with mean *F*_*ST*_ ranging between ~ 0.05 and 0.27 (Figure 1B). Admixture analysis revealed hierarchical population genetic sub‐structure in our data, with K = 5 suggested by the ΔK method (Figure 1E) and K = 10 based on ln(Pr(X|K)) (Figures S6 and S7).

Anadromous and resident populations within each river appeared most closely related to each other, based on genetic distances, rather than clustering together based on migratory/ecotype status (Figure 1C). One of the fish sampled from below the falls in the Ray River (RY_B) clustered close to the neighbouring Gweebarra River (WE_B). At the time of capture, this fish was an adult sea trout and, thus, may have migrated from a nearby river. There was a strong association between geographic and genetic distances among anadromous populations (isolation by distance: Pearson's correlation coefficient *R* = 0.78, *p* = 0.0082; Figure S8) suggesting dispersal and gene flow play a role in structuring genetic variation among rivers. Comparing genomic diversity among anadromous and resident populations revealed consistently reduced genome‐wide genetic diversity (*ϴW*) among the latter (Mean ± SD): resident: 0.0006 ± 0.0006 versus anadromous: 0.0009 ± 0.0007; Wilcoxon test *p* < 0.0001; (Figure 1F). Although both groups had a positive Tajima's D on average for regions across the genome, it was consistently higher in the resident populations (Mean ± SD): resident: 0.757 ± 1.18 versus anadromous: 0.44 ± 1.01; Wilcoxon test *p* < 0.0001; (Table 1), consistent with past population contractions in resident contexts.

### 3.2 Polygenic Adaptive Divergence Associated With AMTs

**FIGURE 2:**
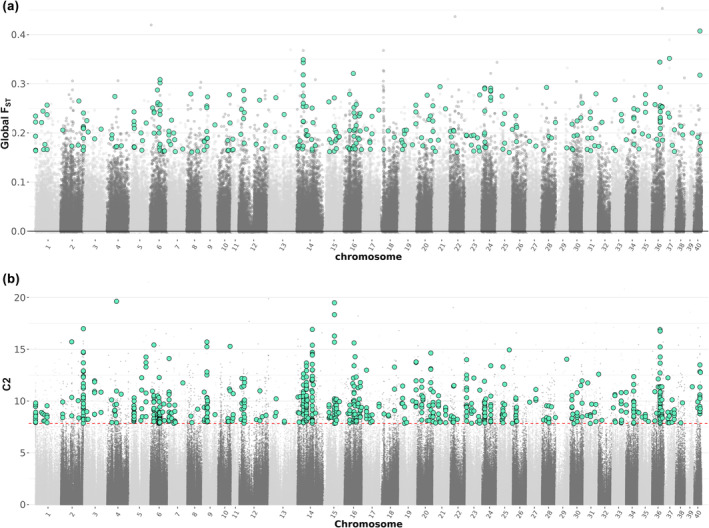
Genome‐wide distribution of outliers that differentiate anadromous and resident populations. (a) Global *F*_*ST*_ calculated in 10 kb non‐overlapping windows, between anadromous and resident groups. (b) Baypass C2 (contrast statistic) identified 765 significant C2 SNPs that overlapped with *F*_*ST*_ outlier regions. The red dashed line indicates the significance level based on the top 1% from pseudo‐observed data (PODs). The blue dots indicate outliers that overlapped between the global *F*_*ST*_ (*n* = 329 windows) and C2 (*n* = 765 SNPs) approaches.

Using an *F*_*ST*_–based genome scan approach to compare anadromous and resident pools, we identified 1385 outlier windows (top 1% *F*_*ST*_ 10 kb windows) putatively related to AMTs. In a complementary approach with Baypass, we found 5253 significant SNPs, of which 765 were located in 329 of the *F*_*ST*_ outlier windows, which we refer to as AMT outliers hereafter. These highly differentiated AMT outlier regions were distributed across the genome (Figure 2; Figure S9) and the number of outliers was correlated with chromosome length (Pearson's correlation coefficient *R* = 0.44, *p* = 0.005).

**FIGURE 3:**
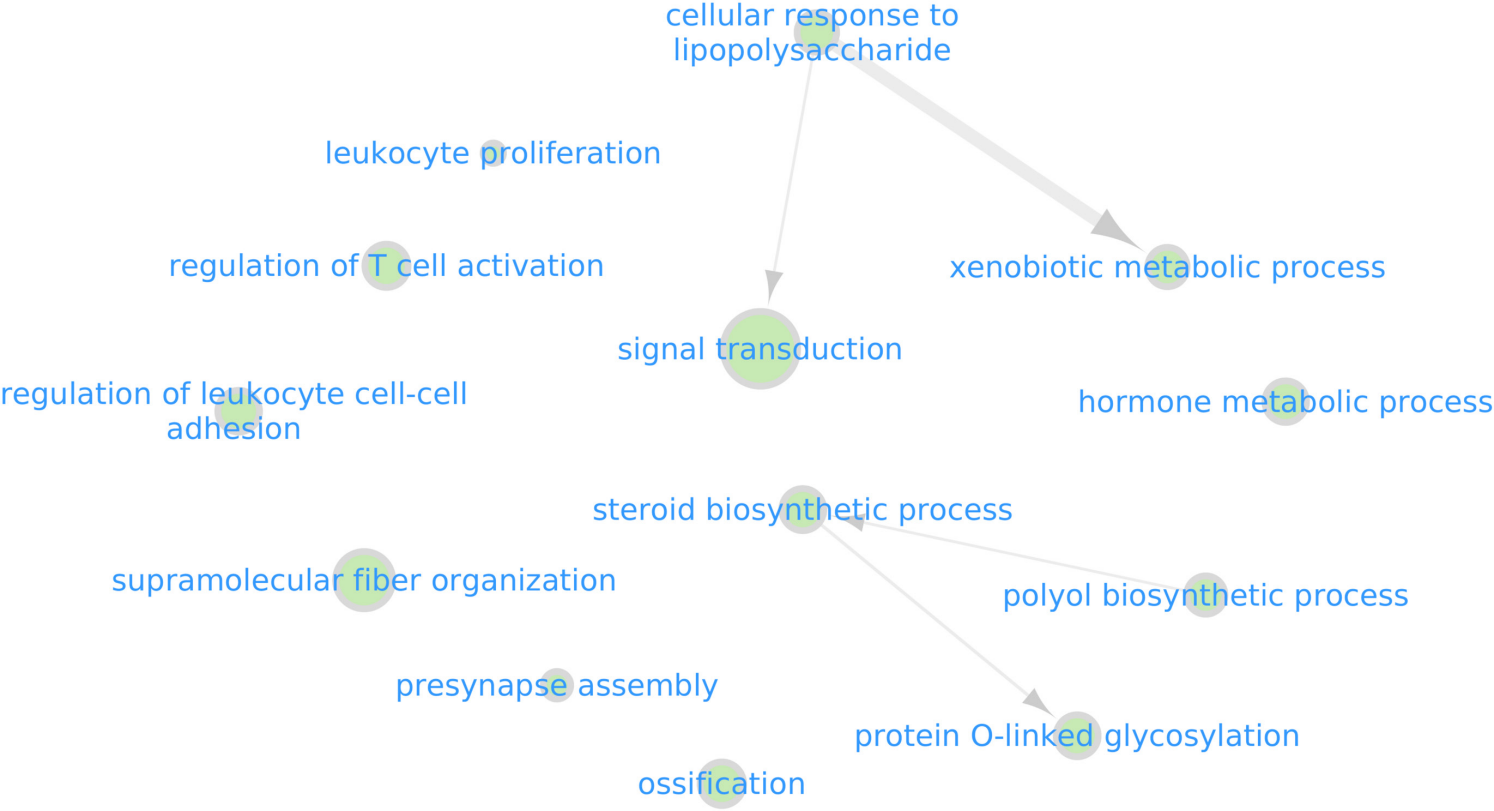
Gene ontology (GO) term enrichment analysis identified biological processes (BP) associated with AMT genes (*n* = 292) based on a node size of 10 (GO terms with *p*‐values < 0.01 included) and visualised using Cytoscape v3.10.1 (Shannon et al. 2003). Candidate genes were enriched for processes, such as hormonal signalling, lipid metabolism, immunity, tissue development and neural function, which could be under differential selection between anadromous and resident populations. Results for all three ontology categories: BP, biological processes; CC, cellular component; MF, molecular function are shown in Figure S12.

Genes associated with AMT windows (*n* = 292 genes, Table S3) were enriched for 14 GO terms (*p* < 0.01), encompassing a broad range of biological processes including energy homeostasis, lipid and steroid metabolism, immunity, neural function and bone development (Figure 3; Figure S12; Table S4). Of our putative AMT‐associated genes (*n* = 292), ten genes overlapped with genes previously identified to be differentially expressed between smolts and residents in *S. trutta* (Wynne et al. 2021) (Table S5). Despite the limited overlap (hypergeometric permutation test with 10,000 permutations: *p* = 0.098), these genes have important functional links to AMTs and are involved in processes such as the control of glucose, lipid, and steroid metabolism (*RNF213*, *ugt2b5*, ENSSTUG00000050426, ENSSTUG00000000821), tissue development and homeostasis (*PTPRK*, *itgab3*, *hey1*).

Functional annotation of the 765 Baypass SNPs that overlapped AMT outliers indicated up to 29 missense mutations occurring within ten annotated genes (*SFMBT1, ora4, cux2b, mtmr3, rack1, zgc:92107, magi3b, dars1, g2e3* and *ESYT2*) and five uncharacterised genes (ENSSTUG00000008474, ENSSTUG00000000693, ENSSTUG00000008787, ENSSTUG00000020251 and ENSSTUG00000030894). Most variants had moderate to low predicted functional effects. Specific annotations of interest included intronic variants associated with genes involved in energy metabolism (*ESYT2, gramd1b, mtmr3, ptprn2* and *idh2*), steroid metabolism (*dhrs1*), immunity and inflammatory response (*mapk14a, txnrd3, itgb2, nod1, rps19, tsc22d1* and *traip*), tissue development (*hmcn2, magi3b* and *sulf2a*), neural development and processing (*TENM2, dip2ba* and *doc2b*) and cellular signalling (*adgrl4, pea15, sestd1, KCNIP4* and *slc8a1b*).

### 3.3 Genetic Diversity Patterns Suggest Signatures of Directional and Balancing Selection

**FIGURE 4:**
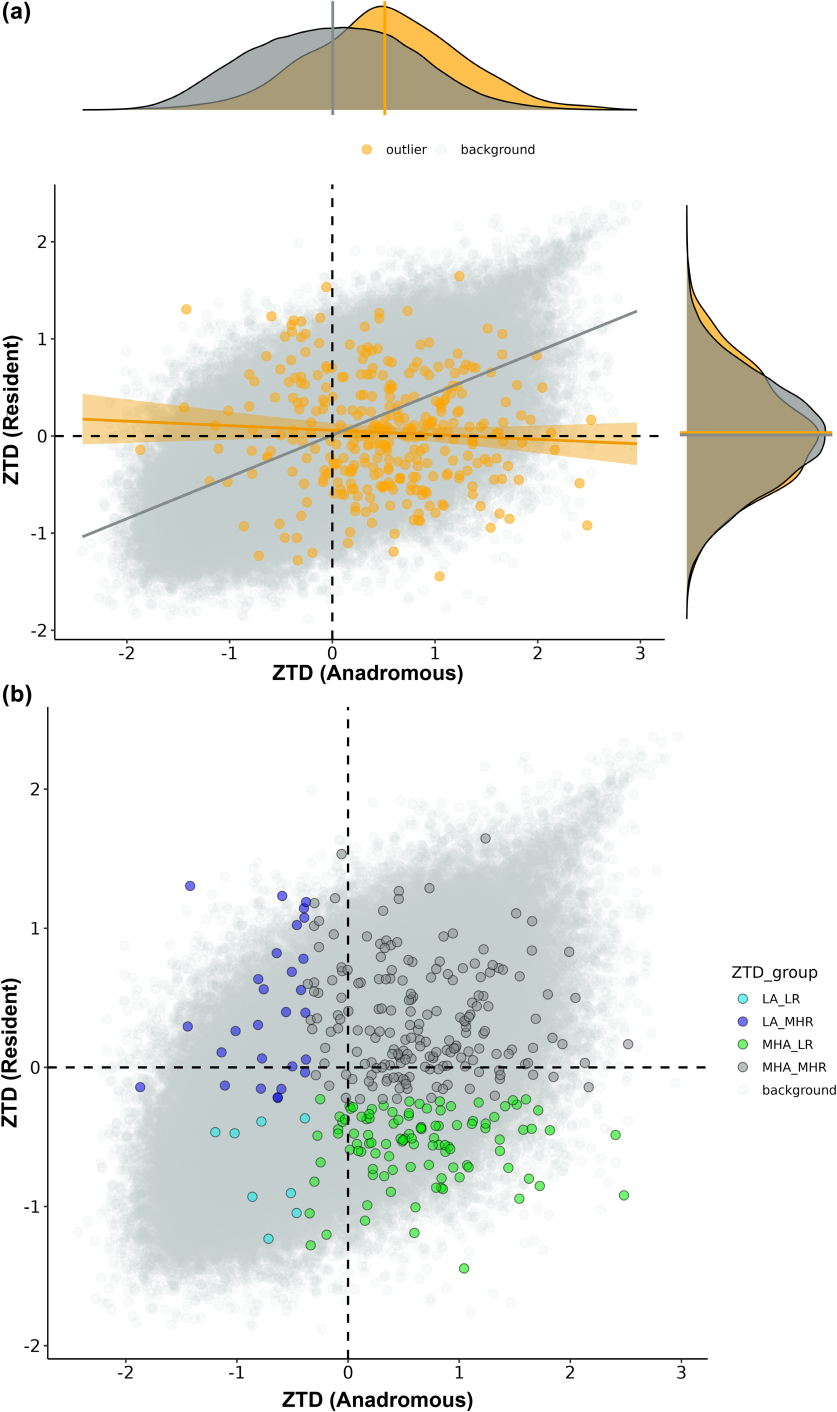
Comparison of Tajima's D (*TD*) (calculated in 10 kb windows and Z‐transformed for each population before getting the overall mean for anadromous and resident groups) for AMT outliers versus the genomic background (non‐outlier windows). (a) Correlation between *TD* among anadromous and resident populations for AMT outliers (orange) and the genomic background (grey). Comparison of the other diversity metrics (pairwise nucleotide diversity and Watterson's Theta) are provided in Figure S3. (b) AMT outliers were subset into tertiles and assigned to one of three groups based on the pattern of *TD* in anadromous and resident groups. Windows with *TD* values in the lower tertile were classified as ‘low’ while windows in the middle and upper tertiles were classified as ‘mid‐high’. Consequently, the four groups were (i) low anadromous and low resident (*LA_LR*); (ii) low anadromous and mid‐to‐high resident (*LA_MHR*); (iii) mid‐to‐high anadromous and low resident (*MHA_LR*); and (iv) mid‐to‐high *ZTD* anadromous and resident (M*HA_MHR*).

Focusing on AMT outliers across different sections of the *ZTD* distribution (lower tertile compared to middle and upper tertiles), we identified a core set of genes potentially under adaptive divergence among the anadromous and resident pools (Figure 4B; Tables S6 and S7A–D). In the *LA_LR* group, eight outlier windows (associated with four genes) were assigned and there was enrichment for one GO term related to hormonal signalling (significant annotated gene: *SSTR2*; Figure S13). In the *LA_MHR* group, 28 outlier windows (associated with 39 genes) were assigned and there was enrichment for one GO term associated with the control of calcium‐dependent exocytosis. In the *MHA_LR* group 105 outlier windows (associated with 102 genes) were assigned and there was enrichment for 11 GO terms related to cholesterol, steroid and hormone metabolism (*CYP27A1*) and immunity (ENSSTUG00000039205, ENSSTUG00000039241). Finally, 188 outlier windows (associated with 176 genes) were assigned to the *MHA_MHR* group and there was enrichment for seven GO terms encompassing carbohydrate, steroid and hormone metabolism, cellular organisation and communication.

### 3.4 Signatures of Balancing Selection

Alternative migratory tactic outliers exhibited higher *ZTD* compared to the background genome in the anadromous pool (mean difference in *ZTD* = 0.51, permutation test *p* < 0.0001) but not the resident pool (mean difference in *ZTD* = 0.02, permutation test *p* = 0.395) (Figure 4A; Figure S14). This finding suggests that some of these regions may experience different selection pressures between both environments, with some under directional selection while others experience relaxed/balancing selection. Considering AMT outliers only, *ZTD* showed no correlation between the anadromous and resident pools (*R* = −0.059, *p* = 0.283). In contrast, the remaining genome exhibited a strong positive correlation (*R* = 0.609, *p* < 0.0001) (Figure 4A). This is in line with some AMT outliers experiencing opposing selection pressures among these two different environments.

In the top 1% of the *ZTD* distribution for the anadromous pool, 12 of the *N* = 6925 10 kb genomic windows (associated with nine genes) were identified as AMT outliers. Notably, nearly half of these outliers were located on chromosomes 16 and 17, while the remainder were distributed across seven other chromosomes (Figure S15). Similarly, within the top 1% of the *ZTD* distribution, four AMT outliers (associated with six genes) were detected in the resident pool (Figure S10). This was more than expected by chance in the anadromous pool (*χ* ^2^ = 23.059, df = 1, *p* < 0.001), but not for the resident pool (*χ* ^2^ = 0.153, df = 1, *p* = 0.696). GO term enrichment analyses for genes linked to AMT outliers in the top 1% of *ZTD* (Table S8) revealed enrichment in the anadromy pool for processes including neurodevelopment and kidney development, as well as lipid metabolism and cellular organisation in the resident pool.

Screening for signals of long‐term balancing selection, in the top 1% of β scores we identified 14 AMT outliers (associated with 17 genes) in the anadromous pool and 10 AMT outliers (associated with 17 genes) in the resident pool respectively (Figure S1; Table S9). This was more than expected by chance in both the anadromous (*χ* ^2^ = 34.865, df = 1, *p* < 0.001) and resident pool (*χ* ^2^ = 13.685, df = 1, *p* = 0.002). GO term enrichment analyses for AMT genes in the top 1% of β scores revealed enrichment for processes involved in immunity, lipid metabolism, oxidative stress and cellular organisation in the anadromous pool and lipid metabolism and cellular organisation in the resident pool.

## 4 Discussion

### 4.1 Key Takeaways

Comparing whole genomes from multiple populations of a facultatively anadromous salmonid species, *S. trutta*, revealed a highly polygenic signal of adaptive divergence associated with contrasting migratory life‐histories. Combining two genome scan‐based approaches, we identified 329 candidate regions distributed across the genome (Figure 2). Examining the genes associated with these regions (*n* = 292 genes 10 kb up or downstream) revealed candidate genes and associated functional processes previously linked to AMTs, such as energy metabolism, reproduction, osmoregulation, neural development and sensory processing (Table 3, Table S4). Within our anadromous populations but not our resident populations, AMT‐associated regions exhibited higher Tajima's D than the genomic background (Figure 4A). This suggests a potential role for balancing selection in maintaining genetic diversity at AMT loci in below‐falls ecological contexts, where both migrants and residents may coexist.

**TABLE 3:** Selection of the most promising candidate AMT genes identified in this study.

Chr	Gene	Function	Previous studies	Trait type
26	*SSTR2*	Hormonal regulation	Differentially expressed in brain of resident vs. migratory rainbow trout (Hale et al. 2016)	Mig‐propensity
31	*dhrs1*	Lipid and steroid metabolism	Gene family linked to adaptive divergence in migratory sockeye salmon ecotypes (Tigano and Russello 2022) and differentially expressed in brains of juvenile resident vs. migratory rainbow trout (Hale et al. 2016)	Mig‐propensity
7	*CERK*	Thermal tolerance, cardiac function, wound healing	Outlier in comparisons of freshwater redband trout (Chen and Narum 2021) and anadromous vs. resident steelhead trout (Willoughby et al. 2018)	Mig‐lifestyle
8	*KCNIP4*	Ion transport	Adaptation to brackish water in Tibetan naked carps (Tian et al. 2023) and mummichogs (Wagner et al. 2017)	Mig‐lifestyle
14	*ora4*	Sensory perception of smell	Outlier in anadromous vs. resident rainbow trout (Hale et al. 2013)	Mig‐lifestyle+
24	*tsc22d1*	Immune response	Immune responses in rainbow trout (Salem et al. 2008) and in Atlantic salmon, infected with *Piscirickettsia salmonis* (Tacchi et al. 2011)	Mig‐lifestyle+
36	*srd5a1*	Reproductive processes	Metabolism of testosterone into progesterone and corticosterone (Martyniuk et al. 2013)	Mig‐lifestyle
2	*cdh23*	Cell adhesion/sensory perception	Adaptation to brackish water in Tibetan naked carps (Tian et al. 2023)	Residency
6	*CDH20*	Cell adhesion	Outlier in comparisons of marine and freshwater sticklebacks (Ferchaud et al. 2014). Gene family involved in alternative migratory behaviour in brown trout (Lemopoulos et al. 2018)	Residency+
16	*SFMBT1*	Growth and development	Growth‐related traits in farmed fish (Yang et al. 2020)	Residency
16	*per3*	Circadian rhythm	Per2 involved in rapid genetic adaptation to novel environments in pink salmon (Sparks et al. 2023)	Residency
16	*grm6b*	Neural function	Gene family involved in migratory behaviour in brown trout (Lemopoulos et al. 2018) and rainbow trout (Hale et al. 2013; Baerwald et al. 2016)	Residency
20	*sestd1*	Cell signalling	Parallel marine‐freshwater divergence in the nine‐spined stickleback (Pungitius pungitius) (Wang et al. 2020)	Residency

*Note:* These 13 genes are a subset of the 292 AMT genes (Table S3) and were selected based on two criteria: (i) belonging to one of the three main *ZTD* groups (see Section 2); and (ii) previously identified as associated with AMTs or have predicted functional links. The final column ‘Trait type’ indicates group assignments based on the pattern of Tajima's D (*ZTD* groups). The three main groups were (i) migratory propensity (*LA_LR*); (ii) migratory lifestyle (*LA_MHR*); and (iii) residency (*MHA_LR*). Cases where genes of interest were also assigned to the fourth *ZTD* group (M*HA_MHR*) are indicated by +.

### 4.2 Polygenic Basis to AMTs in Brown Trout

The polygenic adaptive divergence we detected between migratory and resident populations is consistent with previous studies of salmonid fishes (Hecht et al. 2012, 2013; Kjærner‐Semb et al. 2020; Lemopoulos et al. 2018, 2019; Perrier et al. 2013) and indeed a polygenic basis to AMTs may be expected for several reasons. First, the complexity of migratory behaviour requires the integration of multiple physiological, behavioural and morphological traits, such as changes in osmoregulation, metabolism and life‐history traits. Such complexity is likely to involve multiple genes and regulatory elements (Velotta et al. 2022), and, hence, many loci may contribute to heritability of migration‐associated phenotypes. Second, genotype‐by‐environment interactions are believed to play an important role in migration decisions in salmonids (Ferguson et al. 2019) and other migratory taxa (Liedvogel, Åkesson, and Bensch 2011; Pulido 2011) and multiple QTL distributed across the genome might underpin G x E in general (Des Marais, Hernandez, and Juenger 2013; Green et al. 2014; Johnson, Sotoudeh, and Conley 2022). Third, spatiotemporal variation in selection on AMTs favouring balanced polymorphisms at multiple loci may maintain a polygenic architecture over longer evolutionary scales (Bernatchez 2016; Hedrick 2006; Hohenlohe et al. 2010). These arguments would suggest that simpler genetic architectures for complex traits like AMTs may be the exception, rather than the rule. While major‐effect mutations (e.g., structural variants) can sometimes arise and play an important role in driving patterns of adaptive divergence in migration propensity (Pearse et al. 2019; Arostegui et al. 2019) or other well‐defined life‐history traits (Ayllon et al. 2015; Barson et al. 2015; Hecht et al. 2012; Lemopoulos et al. 2019; Micheletti et al. 2018; Prince et al. 2017; Thompson et al. 2019), any unexplained (‘missing’) heritability may still be attributable to many additional loci (Debes et al. 2021; Sinclair‐Waters et al. 2020). Future studies could enhance our understanding of polygenic selection on AMTs by applying alternative methods such as PicMin (Booker, Yeaman, and Whitlock 2023) and AF‐vaper (Whiting et al. 2022), which allow for the detection of different modes of parallel evolution among rivers, offering a more detailed understanding of the selective pressures at play.

### 4.3 Candidate Anadromy Genes

In this study, we attempted to distinguish between genes associated with migratory propensity versus migratory lifestyle (Table 2). Our logic was that, migration‐propensity loci would experience opposing directional selection pressures (with ‘migration‐increasing’ alleles positively selected in below‐falls contexts but negatively selected in above‐falls contexts), and hence, exhibit reduced genetic diversity (low *ZTD*) in both anadromous and resident groups (*LA_LR*). In contrast, migratory‐lifestyle loci were anticipated to be under directional selection in below‐falls populations and experience relaxed selection in above‐falls populations, resulting in low *ZTD* in the former and mid‐to‐high *ZTD* in the latter (*LA_MHR*).

Among the eight outlier windows categorised as migratory‐propensity (*LA_LR*), two of the four associated genes (*SSTR2* and *dhrs1*) regulate energy metabolism, potentially influencing the decision to migrate. The *SSTR2* gene encodes a receptor for somatostatin, a hormone that plays an important role in regulating energy metabolism and growth (Nelson and Sheridan 2006; Very and Sheridan 2002). Previous studies in rainbow trout have identified *SSTR2* as differentially expressed in the brains of juvenile resident and migratory smolts (Hale et al. 2016). Somatostatin hormones have also been implicated in seawater adaptation in steelhead/rainbow trout (*O. mykiss*) (Poppinga et al. 2007) and coho salmon (*O. kisutch*) (Sheridan, Eilertson, and Kerstetter 1998). The *dhrs1* gene is involved in the metabolism of steroids, retinoids and lipids and other members of the dehydrogenase/reductase SDR family have previously been implicated in adaptive divergence among migratory ecotypes in salmonids. For example, *dhrs7* has been identified as under divergent selection between migratory and resident sockeye salmon (*O. nerka*) ecotypes (Tigano and Russello 2022). Additionally, it has been shown to be differentially expressed in the brains of juvenile resident and migratory rainbow trout (Hale et al. 2016).

The putative migratory‐lifestyle loci (*n* = 28 outlier windows), which exhibited signals of positive selection in the anadromous group and relaxed selection in the resident group (*LA_MHR*), were associated with 39 genes involved in energy metabolism, immunity, osmoregulation, olfaction and reproductive behaviour. The gene *KCNIP4* is a member of the family of voltage‐gated potassium (Kv) channel‐interacting proteins (KCNIPs) that regulate calcium ion transport and has been implicated in salinity adaptation in Tibetan naked carps (*Gymnocypris przewalskii*) (Tian et al. 2023) and mummichogs (*Fundulus heteroclitus*) (Wagner et al. 2017). The gene *tsc22d1* modulates the TGF‐beta signalling pathway and has been implicated in immune responses in *O. mykiss* (Salem et al. 2008) and in Atlantic salmon, *Salmo salar*, infected with *Piscirickettsia salmonis* (Tacchi et al. 2011). Genes from the *TSC22* family have also been implicated in osmotic stress in fish in response to environmental salinity changes (Komoroske et al. 2016; Tse, Lai, and Takei 2011).

Another interesting candidate gene found in our migratory‐lifestyle category was the olfactory receptor gene *ora4*, which plays an important role in homing behaviour (Johnstone et al. 2012). Olfactory receptor genes have previously been linked to adaptive divergence between anadromous vs. resident ecotypes of *O. mykiss* (Hale et al. 2013). Another gene of note, CERK, was associated with putative migratory‐lifestyle outliers in our analysis. The CERK gene is involved in thermal adaptation and cardiac function in redband trout (*O. mykiss gairdneri*) (Chen and Narum 2021) and in wound healing in steelhead trout (Willoughby et al. 2018). This gene may experience distinct selection pressures in anadromous versus resident populations of *S. trutta* due to the differing physiological demands of migratory behaviour.

### 4.4 Candidate Residency Genes

Our third grouping consisted of AMT outliers that exhibited mid‐to‐high levels of genetic diversity in the anadromous group but low diversity in the resident group (*MHA_LR*). Genes associated with this category may be under relaxed or balancing selection in anadromous populations, but positive selection in resident populations, and hence can be thought of as ‘residency’ genes. Such genes included those involved in growth (*SFMBT1*, *RFT1* and *mustn1a*), circadian regulation (*per3*), neuronal development (*grm6b* and *NRXN2*) and sensory perception (*cdh23*). GO enrichment analysis revealed enrichment for processes related to hormone, cholesterol and steroid metabolism (Figure S13). The *per3* gene is part of the clock gene family which act as an internal time‐keeping system and are central in regulating various physiological processes (Leder, Danzmann, and Ferguson 2006; O'Malley, Ford, and Hard 2010; Paibomesai et al. 2010). Resident populations of brown trout situated above waterfalls could experience different environmental conditions, such as differences in light exposure, temperature, flow regimes and food availability, compared to below‐falls anadromous populations and divergent selection on the *per3* gene could play a role in mediating differences in circadian rhythms or activity patterns (Bolton et al. 2021; Sparks et al. 2023).

Genes involved in neural development and function (*grm6b* and *NRXN2*) could also be under positive selection in resident populations. The *grm6b* gene is of particular interest as it is involved in regulating neurotransmission and vision (Huang et al. 2012). Additionally, it belongs to a family of metabotropic glutamate receptors involved in regulating neurotransmission and synaptic plasticity (Ferraguti and Shigemoto 2006), which have previously been implicated in divergence between migratory ecotypes in salmonids. For example, *grm4* was previously found to be a potential target of divergent selection between anadromous and resident *S. trutta* populations (Lemopoulos et al. 2018) and *grm1* has been found to be under divergent selection and differentially methylated between *O. mykiss* migratory ecotypes (Hale et al. 2013; Baerwald et al. 2016).

### 4.5 Balancing Selection on AMT Genomic Regions

A striking feature of our analyses on genetic diversity is that only around 13% of our AMT outliers fell into the *LA_LR* or *LA_MHR* categories. The remaining ~ 87% either exhibited higher diversity in the anadromous group relative to the resident group (*MHA_LR*; ~ 32% of AMT outliers) or similar diversity levels in both groups (*MHA_MHR*; ~ 55% of AMT outliers). Loci falling into these latter two categories are still under putative divergent selection between anadromous and resident populations (otherwise, they would not show up as outliers), yet selection clearly has not driven alternative alleles to fixation in each case as this would have left a signal of reduced diversity in both groups. Balancing selection may, therefore, be at play; indeed, genetic diversity (*ZTD*) was on average higher for our AMT outliers than the genomic background, particularly within the anadromous group. To ensure our findings were not technical artefacts from the pooled genome scan approach or mapping issues, we re‐ran our pipeline to examine river‐specific outliers (local *F*_*ST*_) and identify and remove potential paralogs. The local FST analysis revealed a strong overlap in outliers with the global approach and Tajima's D was consistently higher among both local and global AMT outliers compared to the genomic background, indicating a genuine signal of balancing selection (Section 2 in Appendix S1; Figures S10 and S11). Additionally, there was no evidence of paralog enrichment or duplicated loci affecting our outliers, supporting the observed signal as biological rather than technical noise (Section 1 in Appendix S1; Figures S3 and S4). The overlap in outliers between the local and global approaches validates the global approach's effectiveness in detecting outliers under selection. However, while some key outliers were shared among populations, many were unique to specific population pairs (Figure S10), underscoring the presence of substantial non‐parallel variation that warrants further investigation.

In below‐falls populations, residency may be a viable alternative tactic to migration for certain types of fish (e.g., those in good early‐life condition who do not need to migrate to more productive marine feeding grounds) and some mix of both tactics may be maintained by balancing selection (Dodson et al. 2013; Ferguson et al. 2019). Some of our AMT outliers falling into the *MHA_LR* or *MHA_MHR* categories may thus also influence migratory propensity or influence the performance of fish adopting either tactic. Polymorphism in these genomic regions could be maintained by spatiotemporal variation in selection (Hedrick 2006), heterozygote advantage, frequency dependence or antagonistic pleiotropy, such as sexual antagonism (Rice 1992), which are difficult to distinguish (Bernatchez 2016). We found no evidence supporting sexual conflict linked to survival as a potential driver of balancing selection for AMTs (see Section 3 in Appendix S1; Figure S17; Table S10), but sexual conflict over reproduction remains plausible (Wright et al. 2018). Signatures of possible balancing selection have also been found in migratory ecotypes of Atlantic cod (Karlsen et al. 2013) and in comparisons of marine and freshwater populations of three‐spined sticklebacks (Hohenlohe et al. 2010).

## 5 Conclusions

In summary, we have shown that AMTs likely have a polygenic basis in brown trout, an iconic and widespread species of broad cultural and economic importance and have added to a growing literature characterising the genomic architecture of complex life‐history traits in species capable of facultative migration. It is important to acknowledge that some of the genes we identified in this study may not be involved in AMTs per se but are under divergent selection owing to differences in the abiotic or biotic environments (e.g., differences in food availability, interspecific competition, flow rates and habitat structure). However, these same environmental differences could also drive divergent selection on migratory tactics and the presence of large barriers to upstream migration was the most obvious selective pressure in our study. Our analyses of heterogeneous diversity patterns across the genome point towards balancing selection as a possible mechanism allowing for the coexistence of both migrants and residents in contexts where both tactics are viable, suggesting a fruitful avenue for future research on this topic.

## 6 Benefit‐Sharing

**Benefits generated:** The benefits arising from this research are primarily derived from our sharing of data and findings on public databases.

## Author Contributions

P.A.M., T.E.R., T.J.C. and P.M. conceived the study and experimental design. J.C. and K.P.P. assisted with sample collection. P.A.M. performed the DNA extractions and J.C. performed microsatellite screening. P.A.M. analysed the data with input from T.J.C. and T.E.R. P.A.M. wrote the first draft of the manuscript and all authors discussed the results and contributed to the final version of the paper.

## Conflicts of Interest

The authors declare no conflicts of interest.

## Supporting information


Tables S1–S10

Appendix S1

## Data Availability

**Genetic data:** Whole‐genome resequencing data are available from the European Nucleotide Archive (ENA) (PRJEB72781). **Scripts:** The main scripts used in the analyses are available on DataDryad (DOI: 10.5061/dryad.44j0zpcpz).
