# Supplementary material for: Whole‐Genome Resequencing Reveals Polygenic Signatures of Directional and Balancing Selection on Alternative Migratory Life Histories
Source: Mol Ecol. 2024 Nov 4;33(23):e17538. doi: 10.1111/mec.17538 (PMC11589691; doi:10.1111/mec.17538)
Supplement: Supplementary file 2 — Appendix S1 [file MEC-33-e17538-s001.docx]

**Supplemental Information for:**

# Whole genome resequencing reveals polygenic signatures of directional and balancing selection on alternative migratory life-histories

Peter A. Moran ^1,2, 3*^, Thomas J. Colgan ^1,2,4^, Karl P. Phillips^1,2,5^, Jamie Coughlan ^1,2^, Philip McGinnity ^1,2,6^, Thomas E. Reed ^1,2^

**Table of Contents:**

| 1. **Impact of paralog filtering** | Page 2 |
| --- | --- |
| 1. **Local & Global *F_ST_* approaches** | Page 3 |
| 1. **Sex conflict analysis** | Page 4 |
| **Figure S1** | Page 5 |
| **Figure S2** | Page 6 |
| **Figure S3** | Page 7 |
| **Figure S4** | Page 8 |
| **Figure S5** | Page 9 |
| **Figure S6** | Page 10 |
| **Figure S7** | Page 11 |
| **Figure S8** | Page 12 |
| **Figure S9** | Page 13 |
| **Figure S10** | Page 14 |
| **Figure S11** | Page 16 |
| **Figure S12** | Page 17 |
| **Figure S13** | Page 18 |
| **Figure S14** | Page 19 |
| **Figure S15** | Page 20 |
| **Figure S16** | Page 21 |
| **Figure S17** | Page 22 |
| **Tables** | Moran_2024_Suppl_Tables.csv |

## Impact of Paralogs & Duplicate Loci on Results

**Test for Paralog Enrichment**

To determine if outlier regions of interest are enriched for paralogues, we compared the proportion of single-copy genes versus paralogs found in AMT outlier regions (n=329) with the expected proportion across the entire background genome using a Chi-squared test with Yates' continuity correction. We identified putative single-copy genes and paralogues using resources from Ensembl BioMarts. The single-copy orthologues as based on 1:1 copies found in northern pike, *Esox lucius*. The Chi-squared test yielded a non-significant p-value (X-squared = 0.8312, df = 1, p-value = 0.3619), suggesting no significant enrichment of paralogs within the outlier regions compared to the background. This indicates that our outliers are not primarily driven by the presence of duplicated genes.

**Mean Depth Analysis**

Furthermore, we compared mean read depth across AMT outliers (n=329) and the rest of the genome represented by randomly subsampled windows (n = 2600) of similar size (10kb) to the AMT Outliers. Comparing the median read depth revealed no significant difference between outlier regions and the genomic background (Wilcoxon rank-sum test, p = 0.22), suggesting that duplicated genes do not bias our results, as they would be expected to inflate read depth

**Detect and remove potential paralogs**

To identify paralogous SNPs we first prefiltered our SNPs to remove low frequency variants (MAF < 0.01) resulting in 3,412,042 SNPs retained (originally ca. 10 million SNPs). Following the guidelines on the ngsParalog github (<https://github.com/tplinderoth/ngsParalog>) we used Samtools on the above SNPs to get mpileup. Next, running ngsParalog with the calcLR function on the mpileup output for all samples we found that ca. 4.6% (156,688) of SNPs were potentially paralogous and, thus, removed these sites from our data.

We reran our analyses on this paralog-filtered set of SNPs and compared diversity estimates between AMT regions with the genomic background. Overall, the results were remarkably consistent between the paralog-filtered and unfiltered datasets (Fig. S3). Specifically, when comparing diversity metrics—Tajima’s D, pairwise diversity (π), and Watterson’s Theta (tW)—between outliers and non-outliers, diversity was significantly higher for AMT outliers in both datasets (Wilcoxon test, p < 0.0001) (Fig. S3). This consistency suggests that mapping issues, such as those related to paralogs, are unlikely to influence our results, and we therefore present our findings based on the original dataset

## Comparison of Local vs. Global Approaches

**River-Specific Analyses**: We performed the *F_ST_* analysis for each river separately and examined the concordance between local (per-river) and global (pooled populations) outlier detection methods (details below). Local *F_ST_* outliers were defined as those in the top 5% across three or more rivers. We used this lower threshold for the local *F_ST_* (compared to at the global level) because only six outlier windows were shared among the top 1% across three or more rivers (Fig. S10).

**Integration of *F_ST_* Approaches**: We separately integrated local and global *F_ST_* outliers with significant Baypass C2 SNPs, generating two sets of AMT outliers: local AMTs and global AMTs. We then compared the overlap between these two sets to evaluate the consistency of outlier detection across the *F_ST_* approaches.

**Overlap Analysis**: We analysed the number and percentage of windows/genes that overlapped between the local and global AMT sets. We also compared mean Tajima’s D for global and local AMT outliers in anadromous (A) and resident (R) groups to the genomic background.

**Results**: We found that approximately 38% of local AMT windows and 50% of local AMT genes overlapped with global AMT outliers. Despite the modest overlap, these overlaps were highly significant, as confirmed by hypergeometric permutation tests (10,000 permutations, p-value < 0.001) (Fig. S10). Moreover, we found AMT outliers exhibit significantly higher Tajima’s D irrespective of whether we used the global or local *F_ST_* approach (Fig. S11).

**Conclusion**: These findings indicate that many loci identified by the global *F_ST_* analysis are also outliers in individual rivers, validating the global approach's effectiveness in detecting outliers under selection. Additionally, the higher Tajima’s D among AMT outliers compared to the genomic background suggests that the signal of balancing selection is genuine and not an artifact of the genome scan approach.

## Sex Conflict Analysis

**Intro**

Sexual conflict between AMTs might occur because alleles conferring increased migratory propensity might be positively selected in females but negatively selected in males (Fleming & Reynolds, 2004). In theory, sexual conflict can favour the long-term maintenance of balanced polymorphisms, although this is not guaranteed and the effects on genetic diversity may be transient or weak (Flintham et al., 2023).

**Methods**

**Testing for Sexual Conflict in AMT Outliers**

To determine if AMT outliers experience sexual conflict due to viability selection, we calculated inter-sex *F_ST_* (Kirkpatrick & Guerrero, 2014) using the joint site frequency spectrum for males and females within anadromous and resident populations for each river separately. Elevated inter-sex *F_ST_* compared to the background genome would indicate sexual conflict over viability. We examined the relationship between inter-sex *F_ST_* and *ZTD* for AMT outliers in the upper *ZTD* tertile versus lower tertiles.

**Results**

For AMT outliers in the upper *ZTD* tertile, there was no significant association between inter-sex *F_ST_* and *ZTD* in either the anadromous or resident pool (Fig. S17). This suggests no evidence of sexual conflict over viability for these outliers, though sexual conflict over reproduction remains a possibility (Wright et al., 2018).


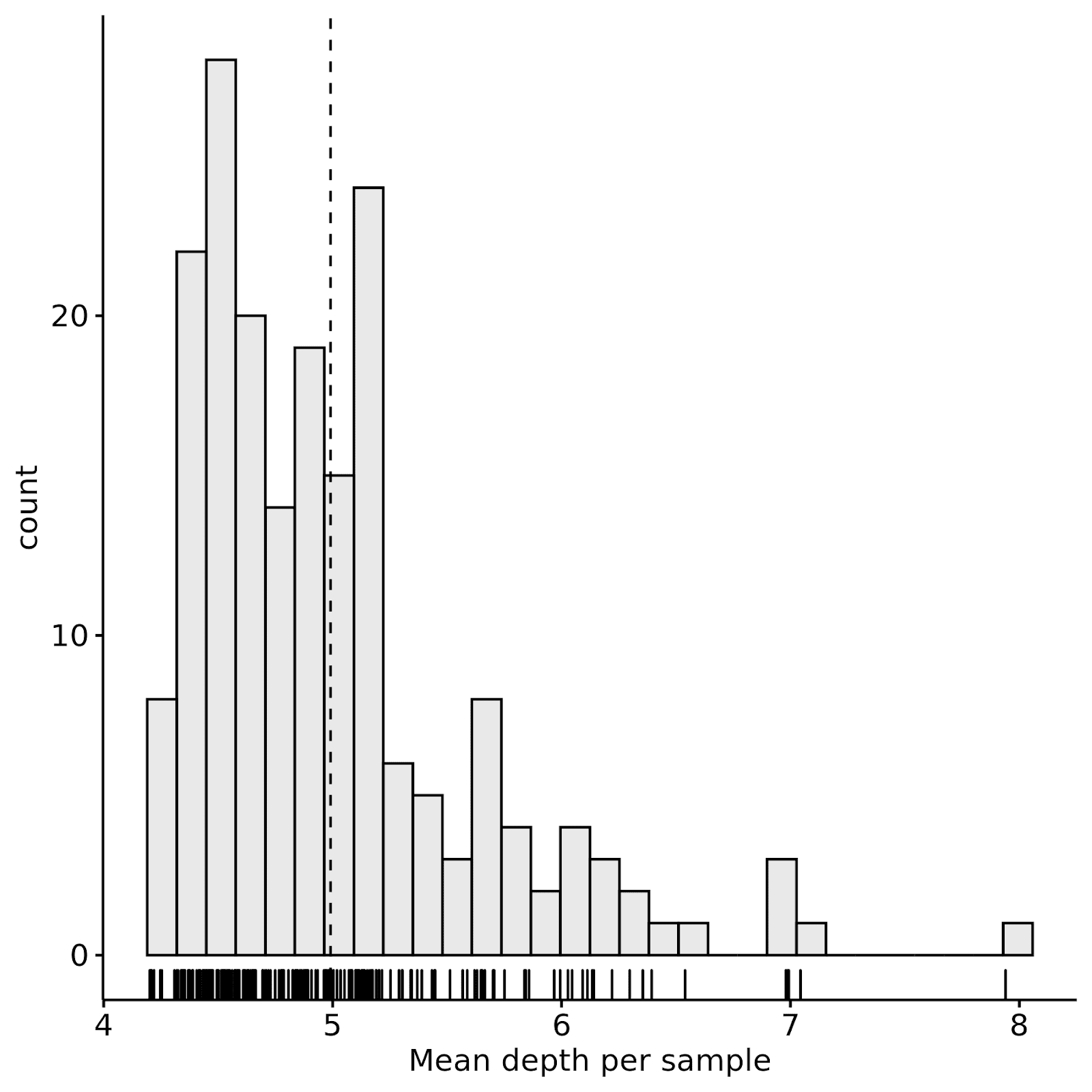


**Figure S1.** Average sequencing depth per sample after initial quality filtering (using Fastp). Mean coverage across all samples was 4.99X indicated by the black dotted line.


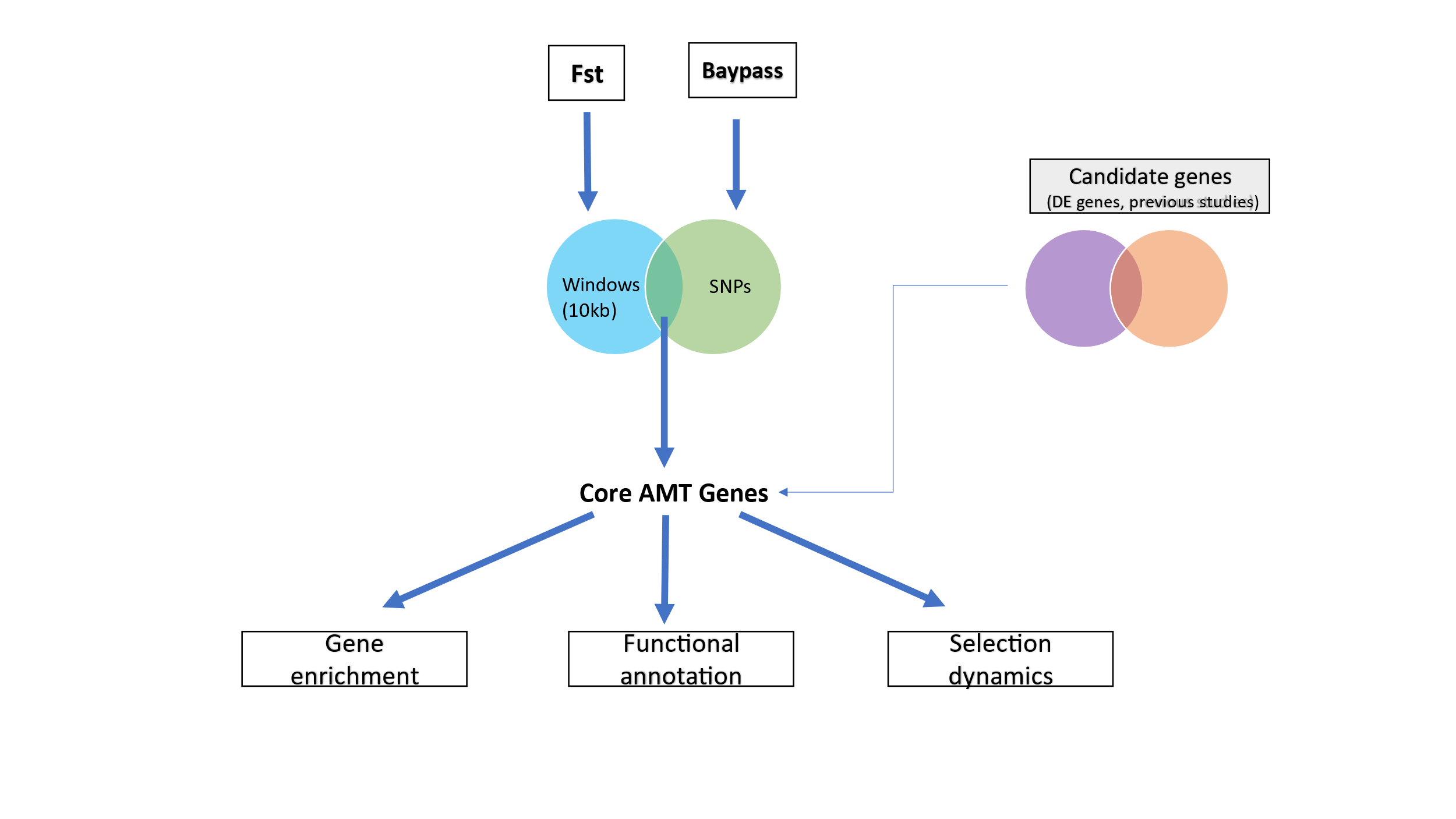


**Figure S2.** Overview of the analysis pipeline for identifying genes and processes associated with alternative migratory tactics (AMTs).


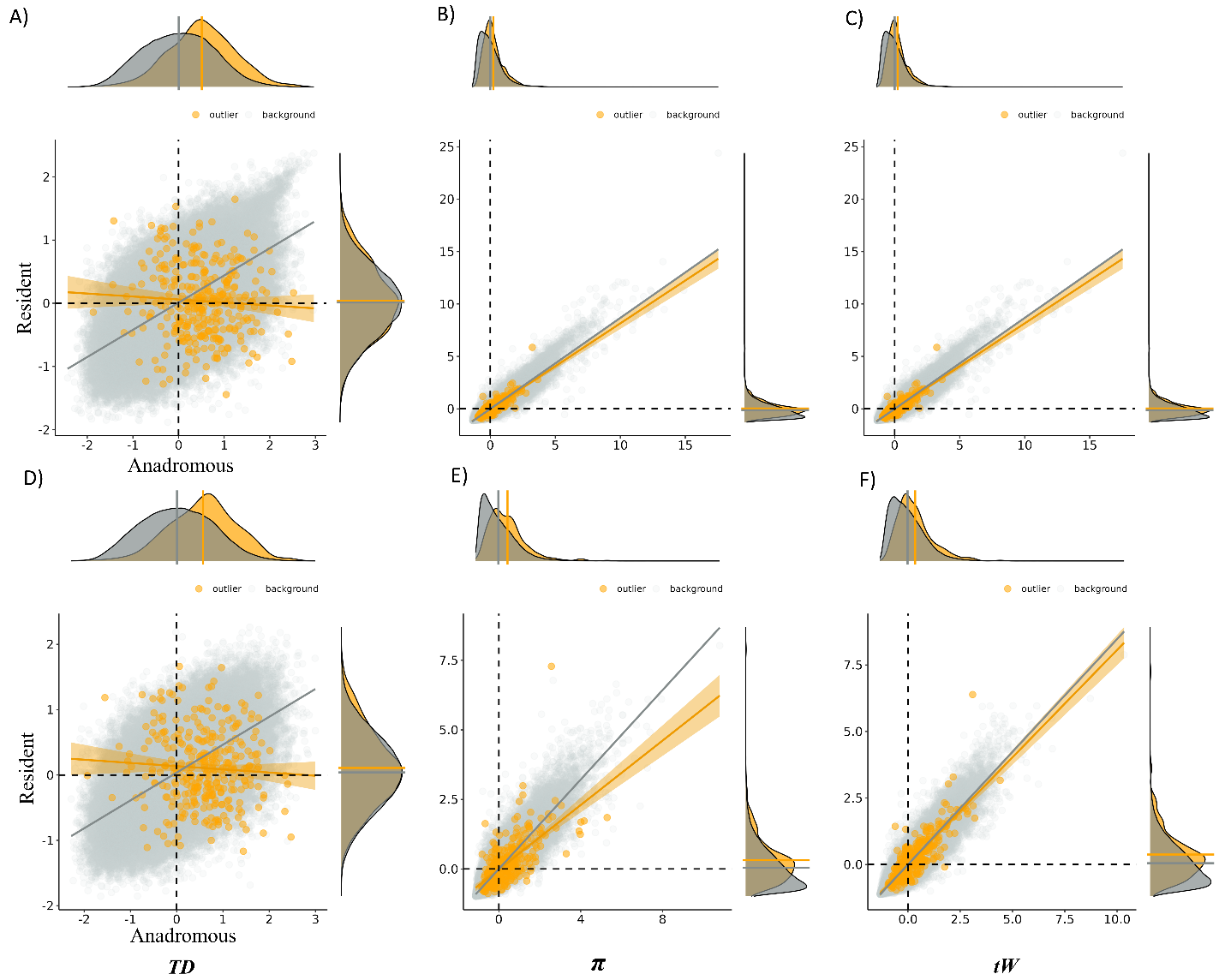


**Figure S3.** Comparison of diversity statistics (10kb windows) for AMT outliers (yellow) versus genomic background (non-outlier windows) calculated for resident and anadromous pools: **A)** Tajima’s D **B)** Pairwise diversity (π) **C)** Watterson’s Theta (*tW*). The second row (D-F) shows the same diversity statistics but for data filtered to remove potentially paralogous SNPs using ngsParalog.


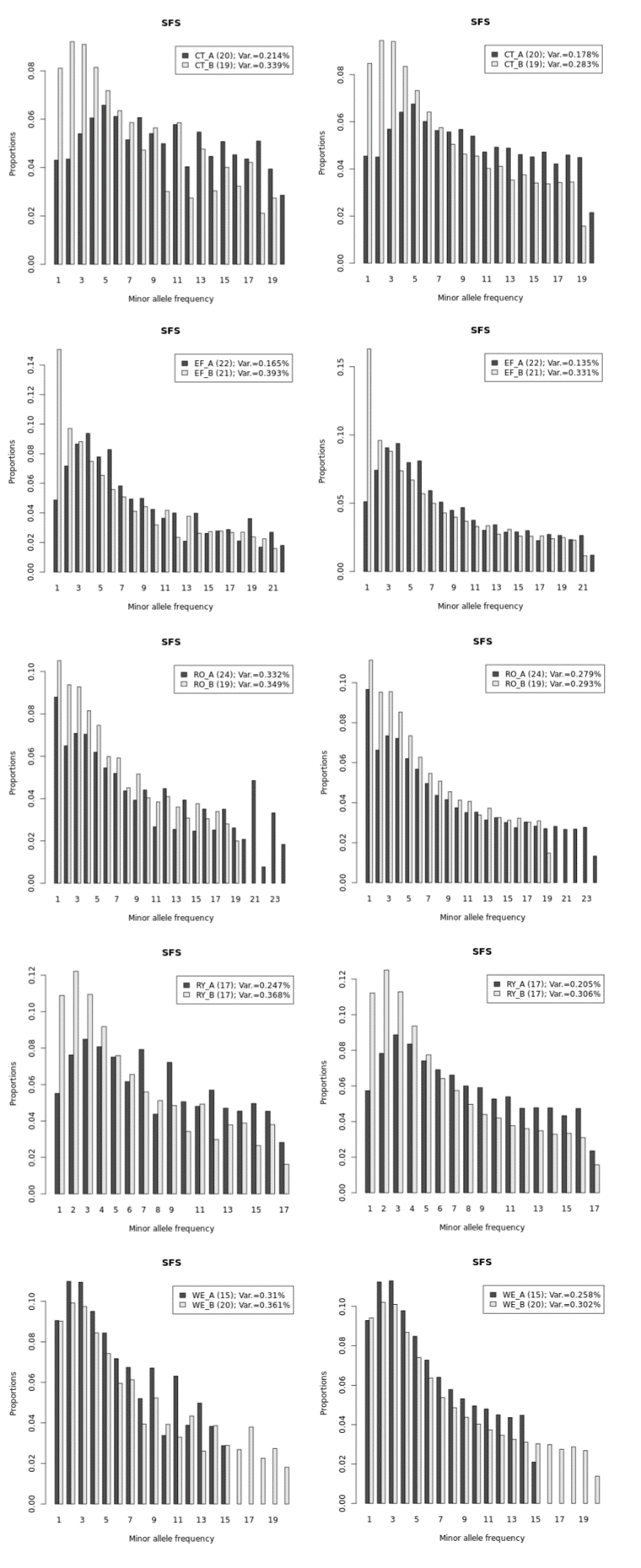


**Figure S4.** Comparison of paralog filtering on the folded site frequency spectrum (SFS). Populations are ordered in geographic order (CT, EF, RO, WE, RY) with anadromous (below-falls (B)) populations indicated by light grey bars and resident (above-falls (A)) populations by dark grey bars. Column on the left presents unfiltered data and on the right the corresponding ngsParalog filtered data (details on ngsParalog filtering above).


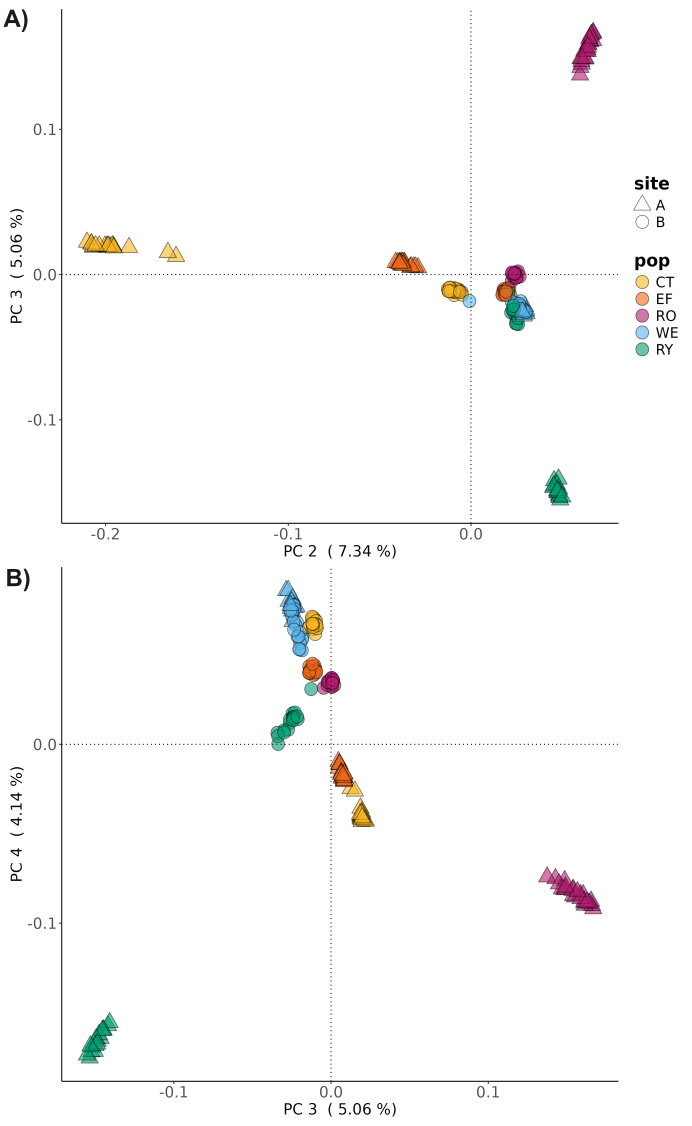


**Figure S5.** Principal component analysis (PCA) showing (**A**) the distribution of genomic variation on PC2 - PC3 and (**B**) PC3 – PC4.


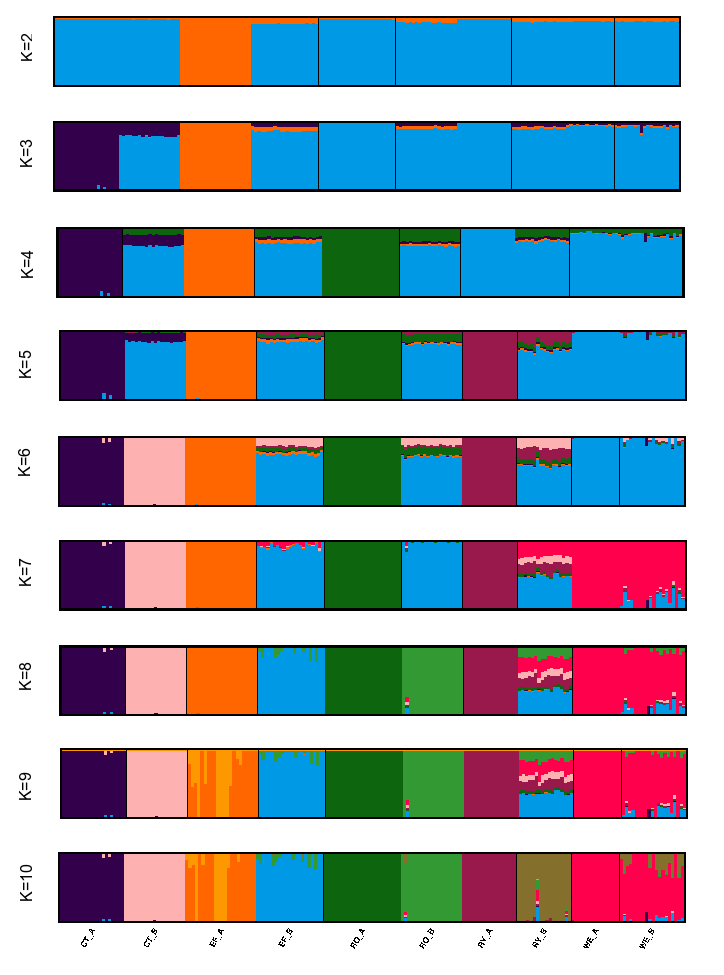


**Figure S6.** Clumpak admixture plots for K 2-10 for all populations (194 samples) based on results from NGSadmix. Each column indicates an individual sample and populations are separated by black lines. Note colours do not correspond to those used in Figure 1 of the main text.

**
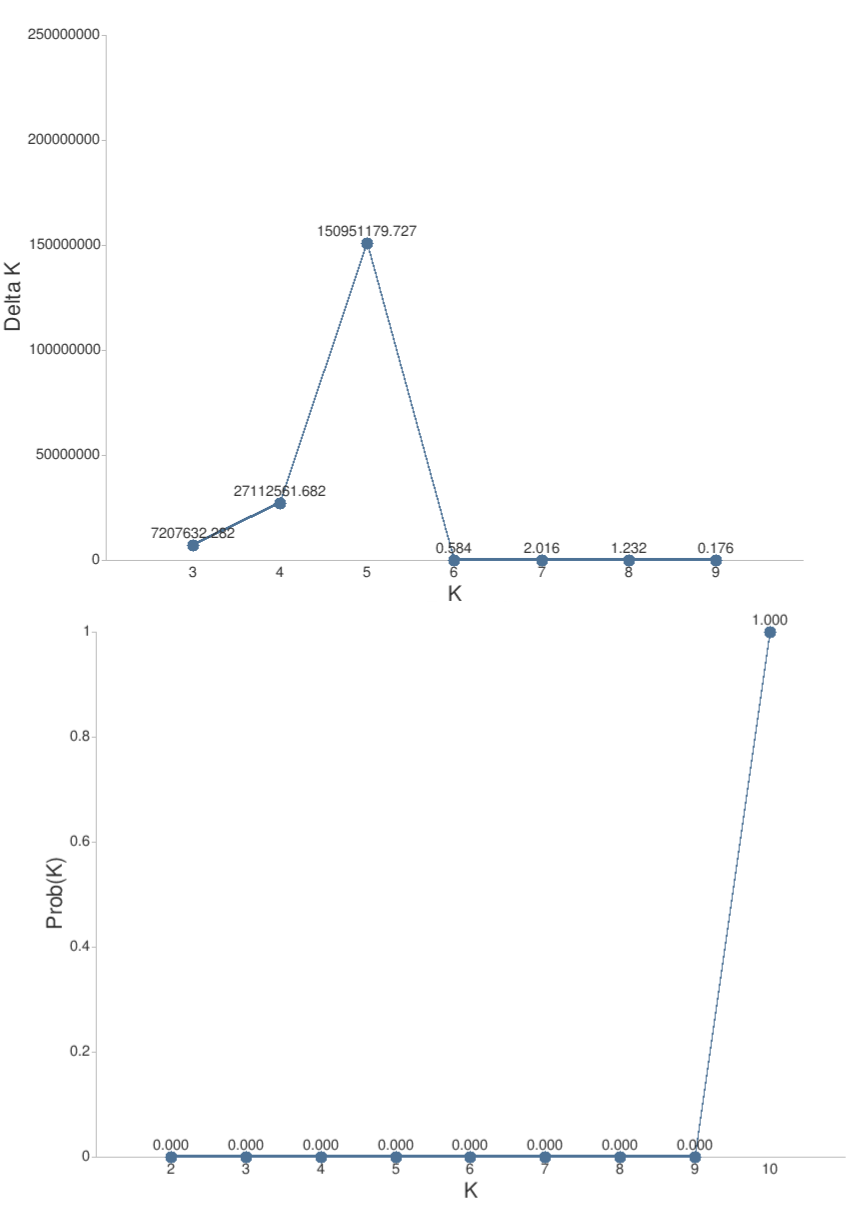
**

**Figure S7**. Best K estimated using Delta K (Evanno et al., 2005) and the mean posteriori probability likelihood (ln(Pr(X|K)) (Pritchard et al., 2000) both calculated and plotted using Clumpak (Kopelman et al., 2015).


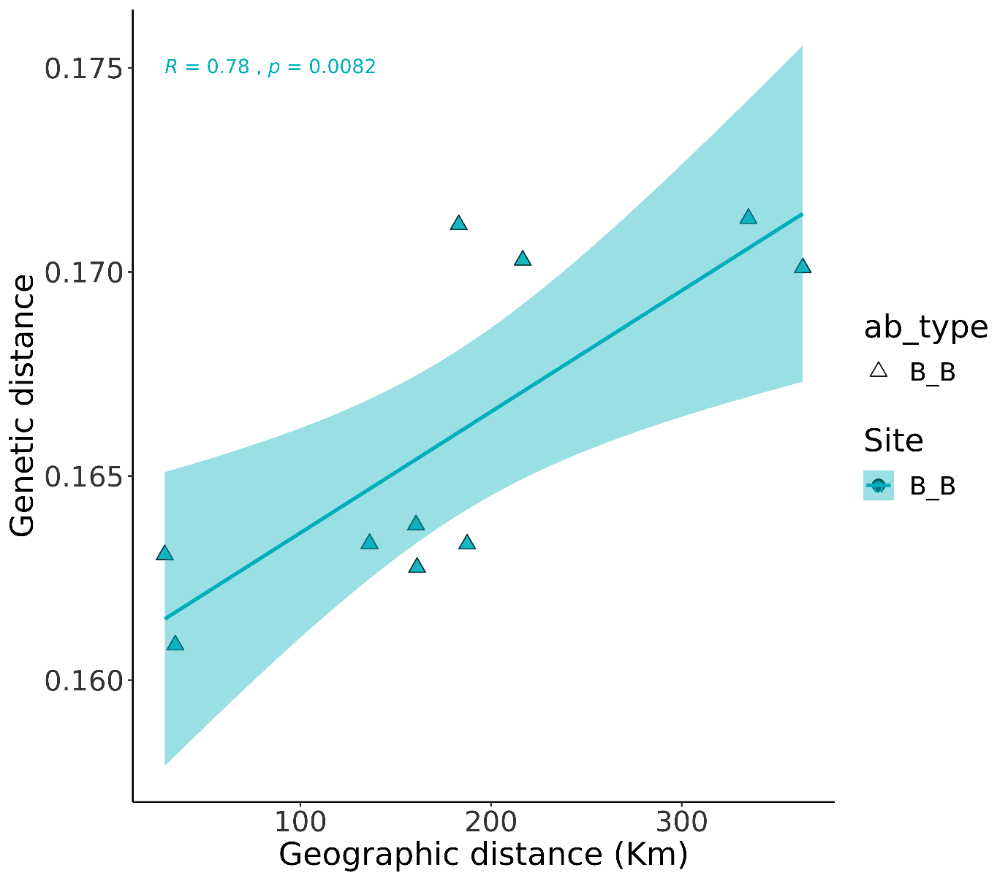


**Figure S8.** Genetic distance and geographic distance among anadromous (below-fall (B)) sites. Genetic distances calculated based on genotype probabilities in ngsDist.


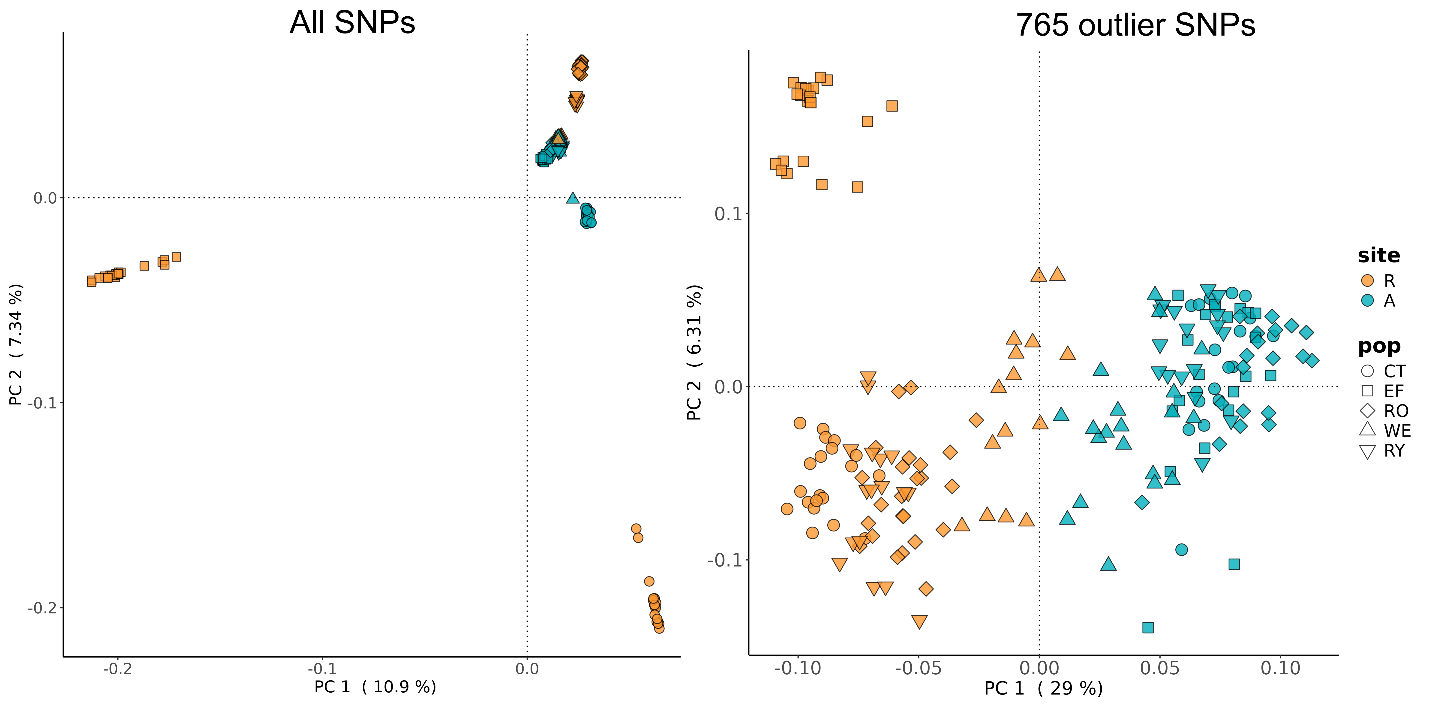


**Figure S9**. **A)** PCA based on all ca. 9 million SNPs. **B)** PCA based on Baypass C2 SNPs (n=765) that overlapped *F_ST_* outlier windows indicating differentiation between anadromous and resident groups.


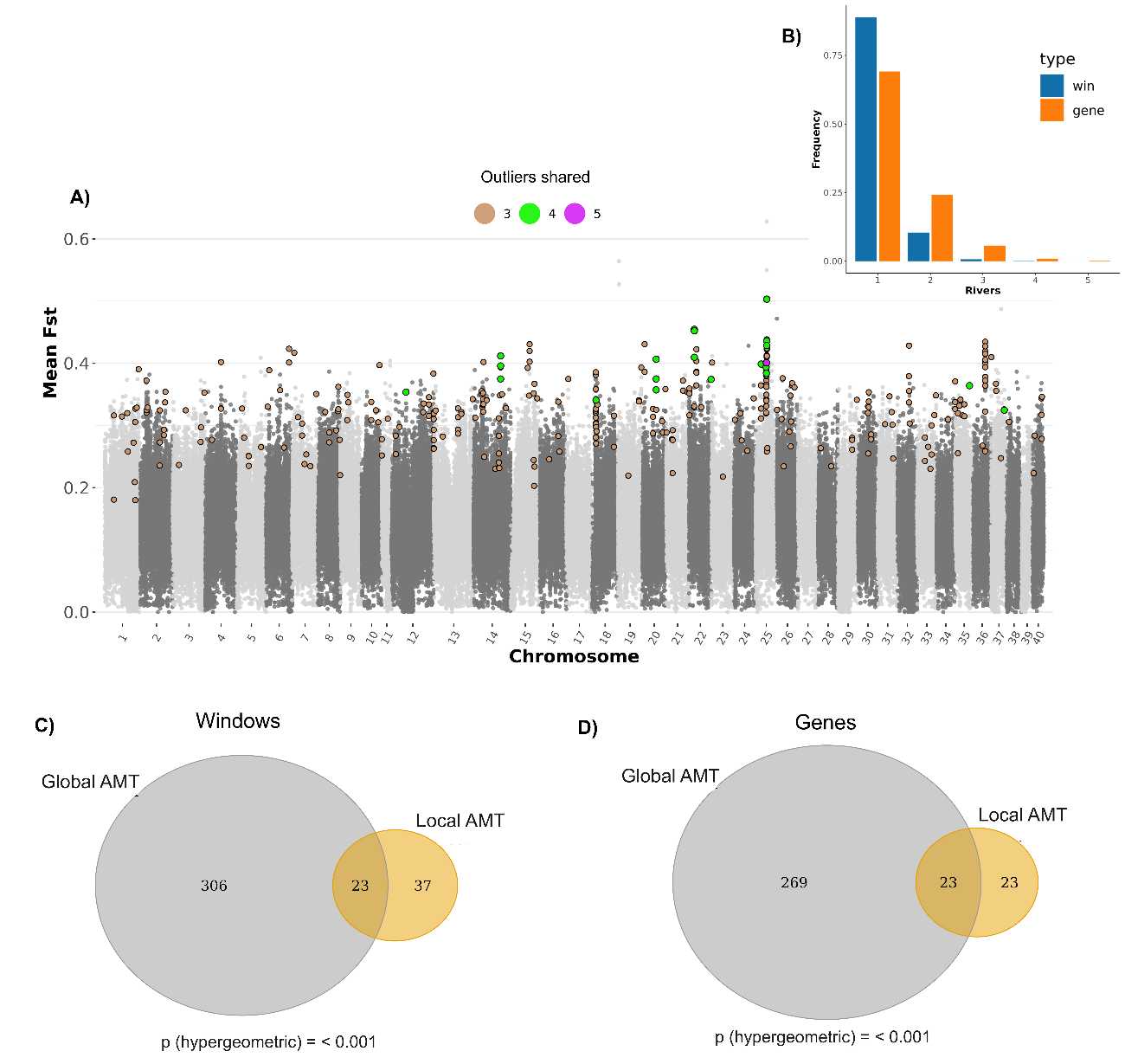


**Figure S10.** Genome-wide differentiation (*F_ST_*) among the anadromous and resident populations showing the level of overlap of outlier windows and outlier genes using local (per-river *F_ST_*) or global (pooled anadromous and resident) approaches. **A)** Manhattan plot showing mean local *F_ST_* (non-overlapping 10kb windows) and distribution of outlier windows across the genome. Outlier windows were defined per-river as those in the top 5%. Each point represents mean *F_ST_* per 10kb window and the highlighted points (legend) represent outliers shared across >=3 rivers. The colors differentiate the level of sharing: brown for outliers shared by 3 rivers, green for those shared by 4 rivers, and purple for the single window shared by all 5 rivers. **B)** Proportion of outlier windows and genes shared across the five rivers. Genes within 10kb (either upstream or downstream) of an outlier window (top 5%) were assigned as outliers. **C-D)** Integrating global and local *F_ST_* outliers with Baypass C2 outliers resulted in two AMT outlier sets. We examined the overlap of Global and Local AMT overlap based on **C)** windows (10kb) and **D)** genes (10kb). Significance of overlap was tested using hypergeometric permutation test with 10,000 permutations. Overall, there was greater sharing of genes than windows on both the per-river level and also among the global and local AMT outliers.


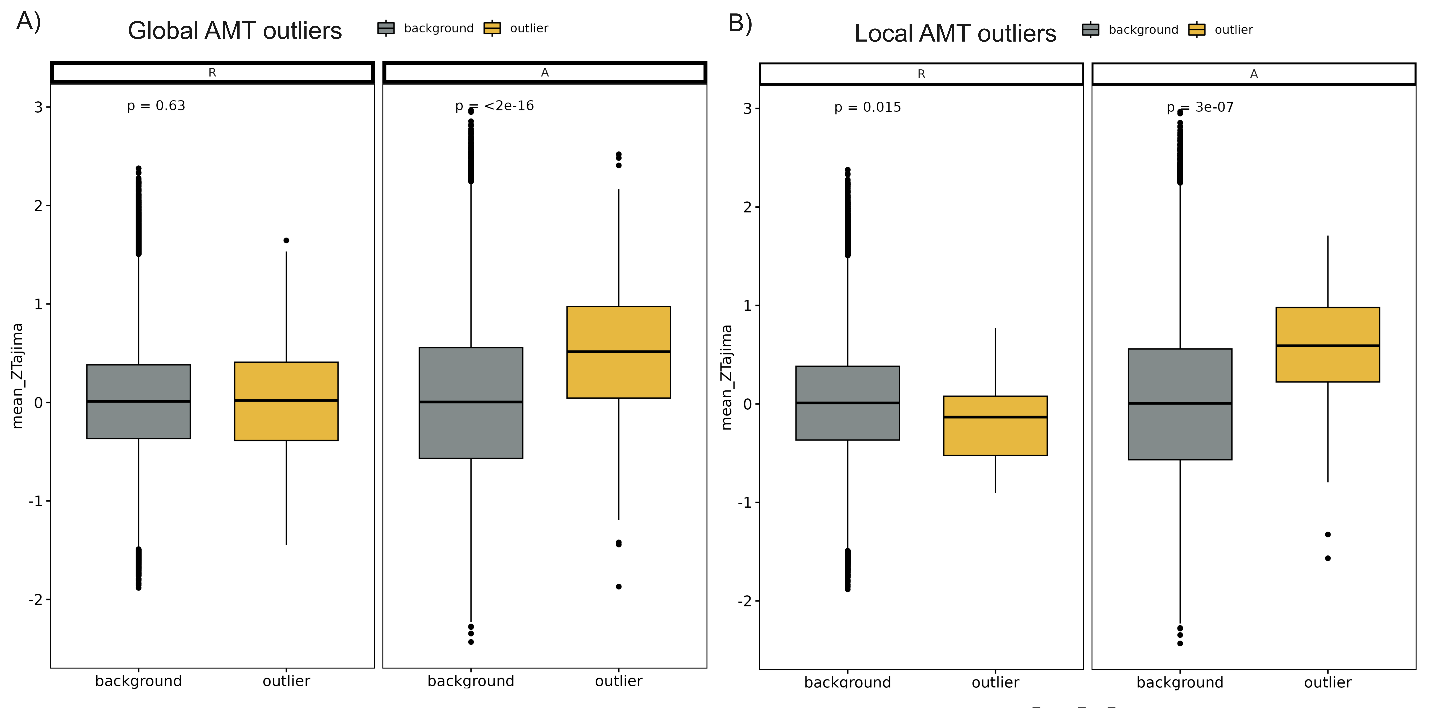


**Figure S11.** Comparison of Tajima’s D for **A)** Global AMT outliers and **B)** Local AMT outliers in anadromous (A) and resident (R) groups. Global and local refers to the *F_ST_* approach used; global (pooled populations) and local (per-river). Overall we find higher Tajima’s D for our AMT outliers, in the an irrespective of whether we used a global or local *F_ST_* approach.


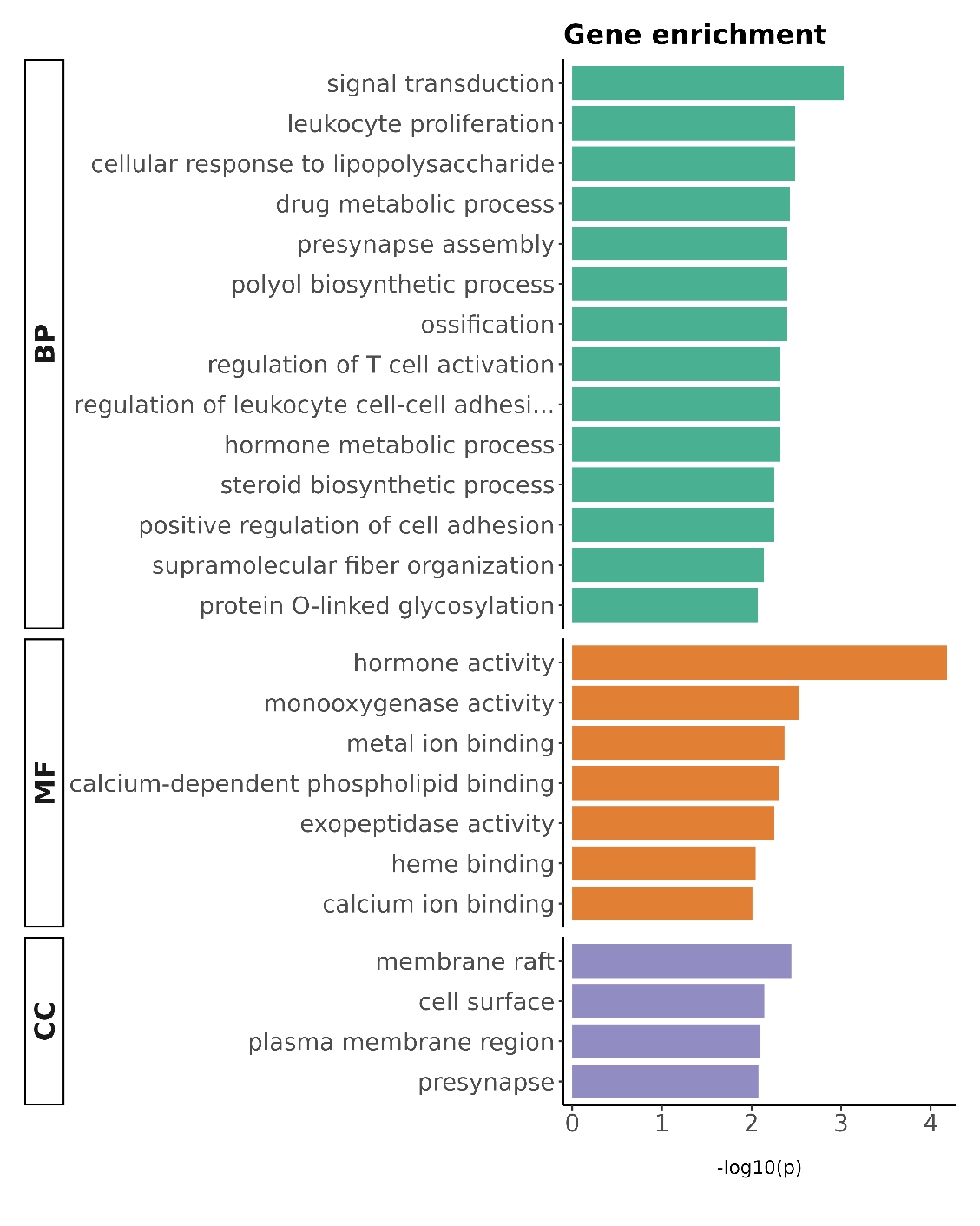


**Figure S12.** Gene enrichment analysis for AMT genes (n=292) based on a node size of 10 (p-values <0.01), indicates genes involved in a variety of processes such as hormonal signaling, lipid metabolism, immunity, osmoregulation (ion transport across membranes), tissue development and growth. Results for all 3 ontology types: Biological processes (BP), Molecular function (MF), and Cellular component (CC) are shown.


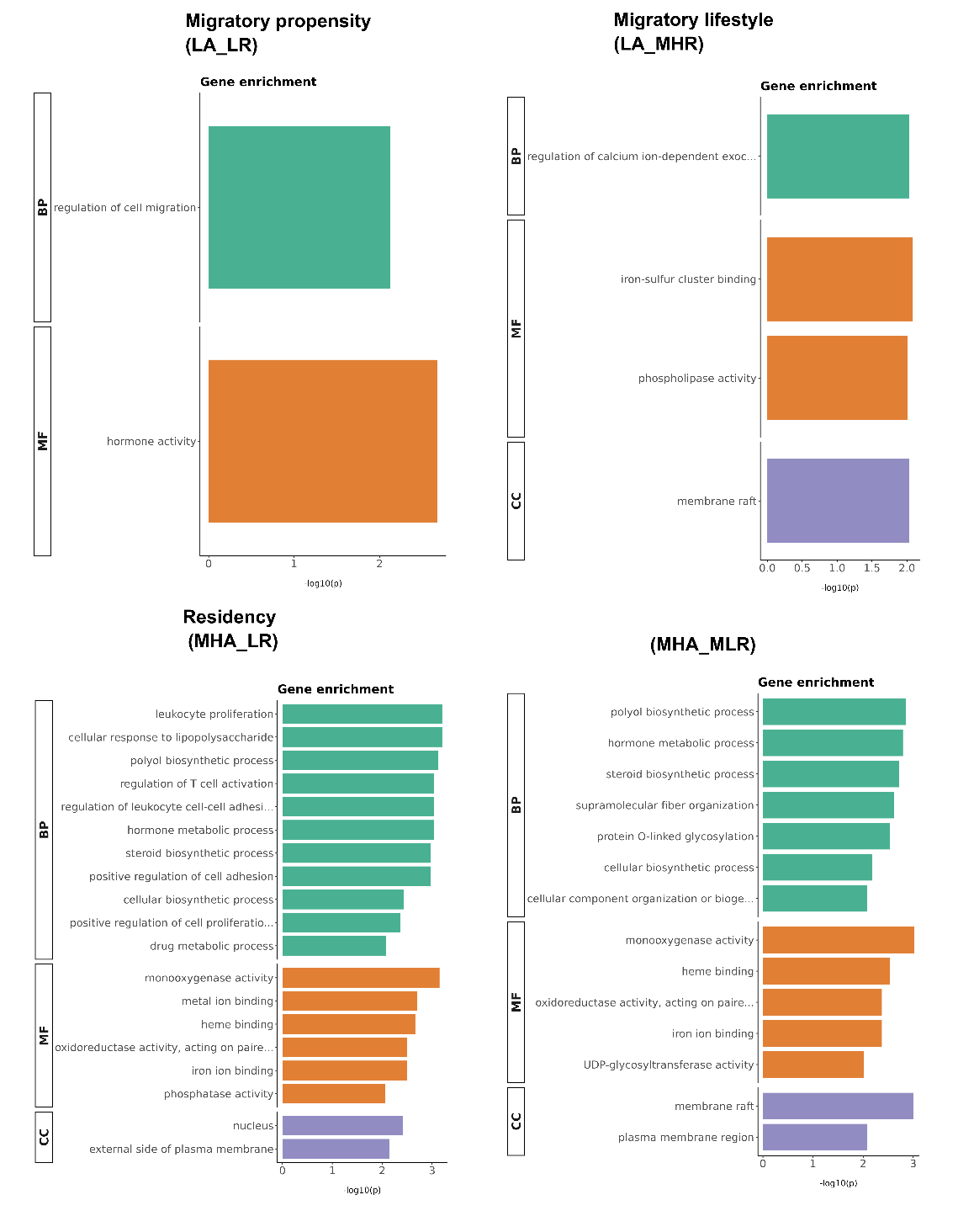


**Figure S13.** Gene enrichment results (node size=10, weight fisher p-values < 0.01) for genes assigned to the different migratory trait type categories based on Tajima’s D in both anadromous and resident populations.


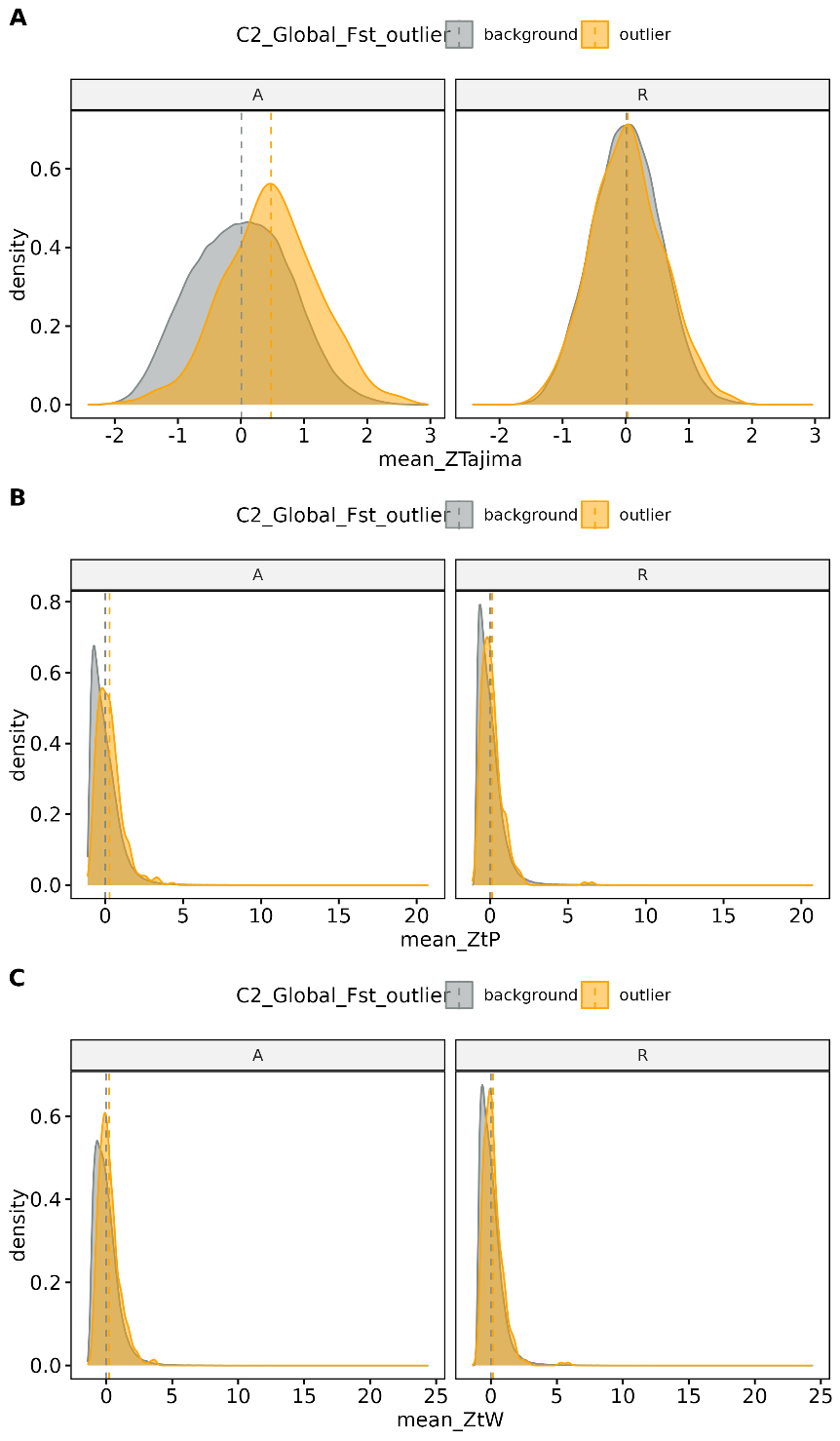


**Figure S14.** Comparisons of diversity statistics for **A)** Tajima’s D, **B)** pairwise diversity (π), **C)** Watterson's theta (ϴW). AMT outliers are coloured in yellow and non-outliers in grey (all other genomic windows) for anadromous (A) and resident (R) pools.


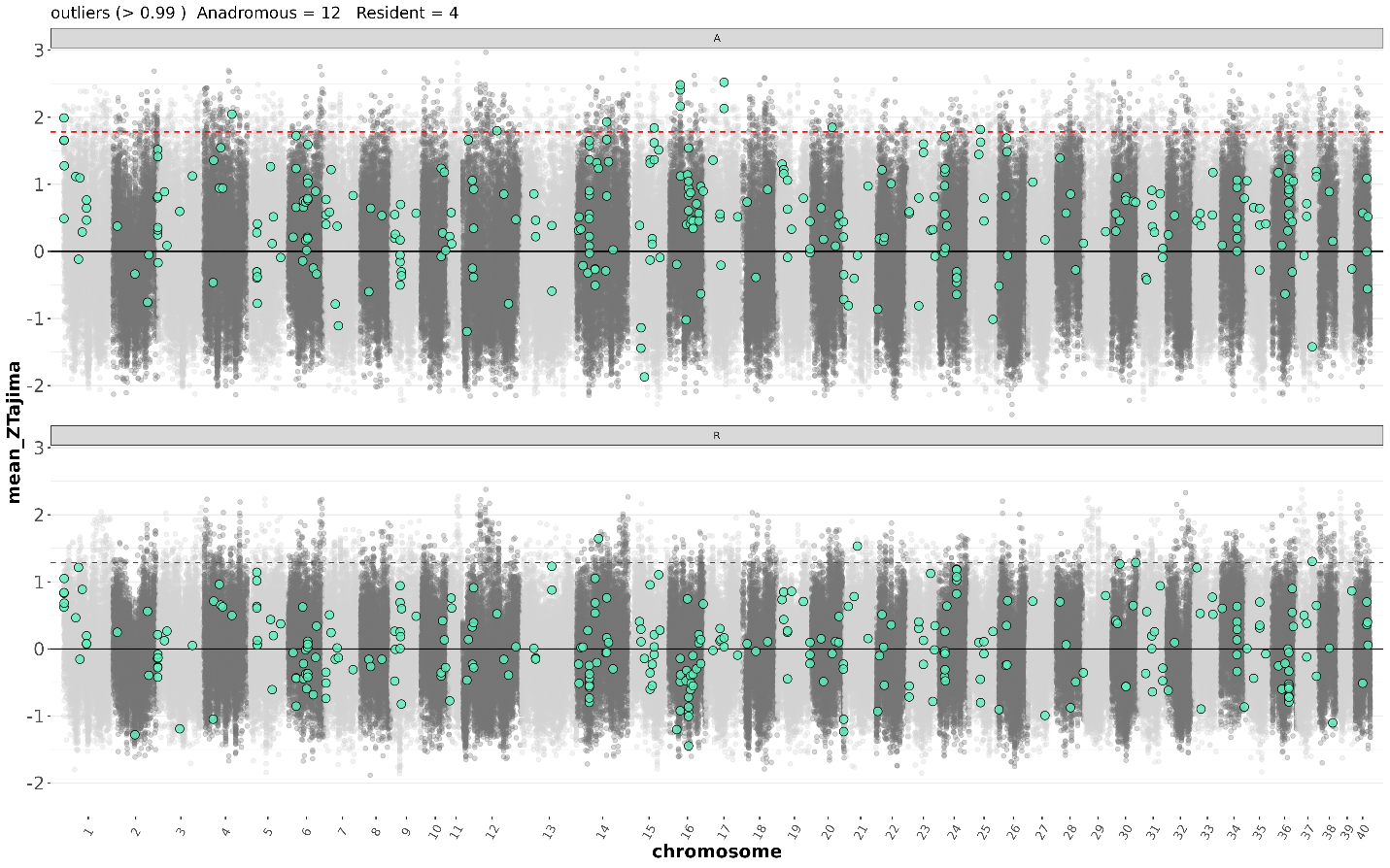


**Figure S15.** Mean Tajima’s D (Z-transformed) calculated in 10kb windows for anadromous (A) and resident (R) groups. AMT outliers are highlighted in green. The red dashed line indicates the 99^th^ percentile based on the genome-wide Tajima’s D (Z-transformed).


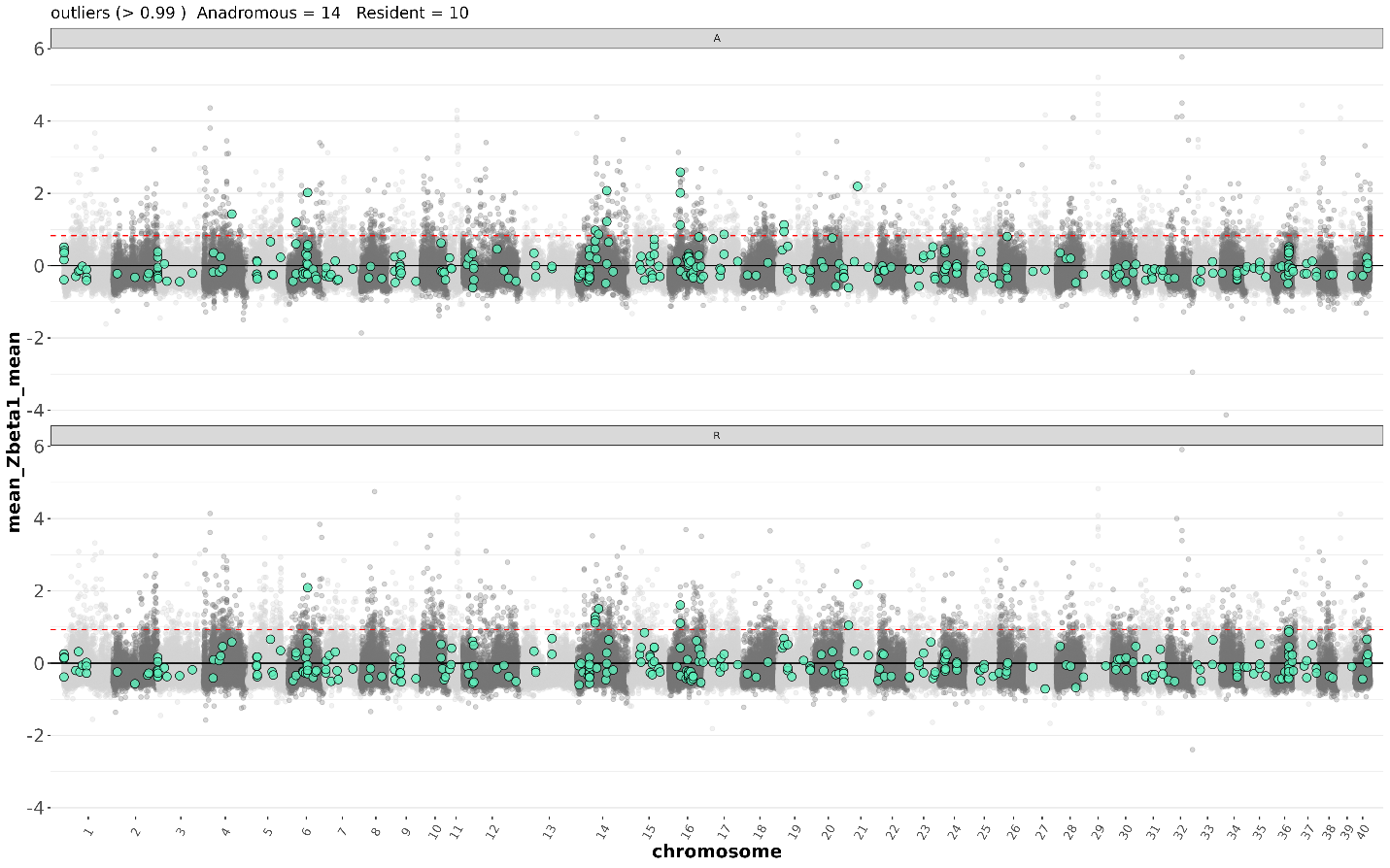


**Figure S16.** Mean β (Z-transformed) calculated in 10kb windows for anadromous (A) and resident (R) groups. AMT outliers are highlighted in green. The red dashed line indicates the 99^th^ percentile based on the genome-wide mean β (Z-transformed).


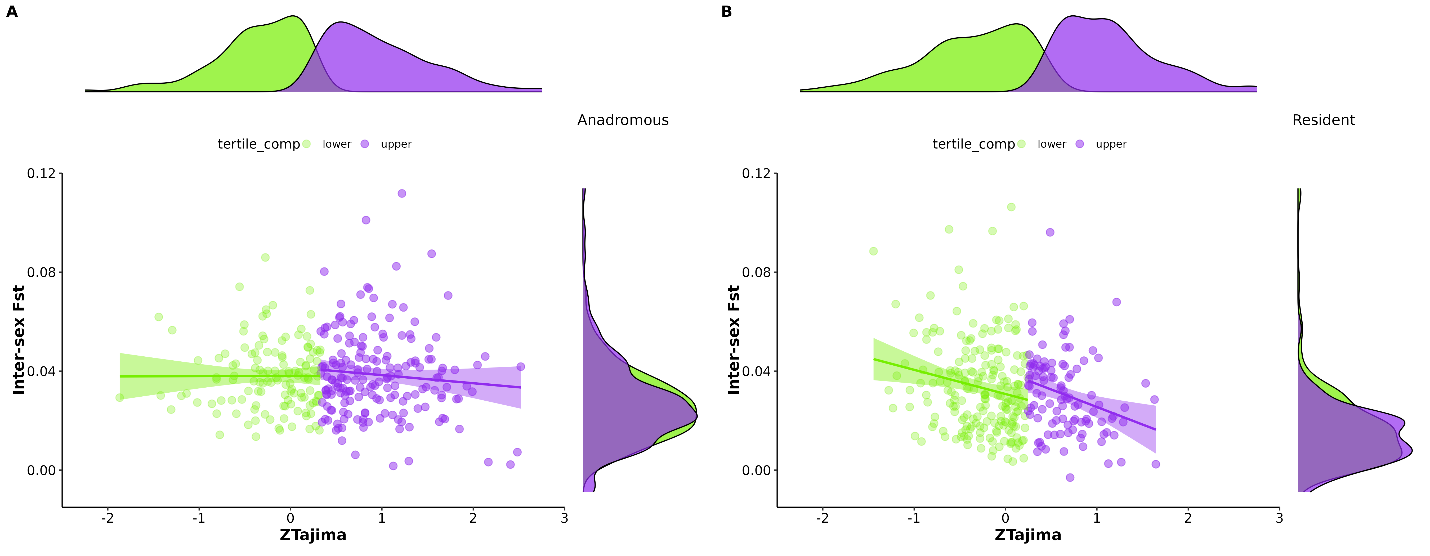


**Figure S17.** Comparing intersex *F_ST_* for our AMT windows upper tertile (colored in purple) and lower tertiles (colored in green) among **A)** anadromous and **B)** resident populations.

## Tables

Supplementary tables are provided on dryad (https://doi.org/10.5061/dryad.44j0zpcpz) and captions are below:

**Table S1.** Per sample summary of raw read quality and predicted coverage based on genome length.

**Table S2.** Global summary of read mapping (clean and filtered reads from fastp) based on Qualimap and multiQC reports.

**Table S3.** Genes (n=292) within 10kb up or downstream of AMT outliers (regions of overlap for *F_ST_* windows and C2 SNPs). Chromosome name (chr), gene name, ensemble gene id, gene start and end positions, distance from outlier windows to gene (bp, 0=range overlap), and gene description.

**Table S4.** GO term enrichment results for 292 AMT genes. Go terms with p<0.01 (weight Fihser i.e. not padjusted) shown.

**Table S5.** Genes that overlapped between putative AMT genes (n=292) with differentially expressed (DE) genes in smolts vs. non-smolts (Wynne et al., 2021).

**Table S6.** The number of AMT outlier windows (n=329) and associated genes (n=292) assigned to one of four groups based on TD. Note nine genes were assigned to more than one *ZTD* group.

**Table S7.** Genes associated with (within 10kb up or downstream) AMT outliers assigned to the four *ZTD* groups. Columns indicate chromosome name (chr), gene name, ensemble gene id, gene start and end positions, distance from outlier windows to gene (bp, 0=range overlap), and gene description.

**Table S7.A)** Genes (n=4) associated with (within 10kb up or downstream) AMT outliers assigned to the *ZTD* *LA_LR* group (lower tertile for both anadromous and resident pools).

**Table S7.B)** Genes (n=39) associated with (within 10kb up or downstream) AMT outliers assigned to the *ZTD* *LA_MHR* group (lower tertile for anadromous pool versus middle and upper tertiles for resident pool).

**Table S7.C)** Genes (n=102) associated with (within 10kb up or downstream) AMT outliers assigned to the *ZTD* *MHA_LR* group (middle and upper tertiles for anadromous pool versus lower tertile for resident pool).

**Table S7.D)** Genes (n=176) associated with (within 10kb up or downstream) AMT outliers assigned to the *ZTD* *MHA_MHR* group (middle and upper tertiles for anadromous and resident pools).

**Table S8.A-B)** Genes associated with (within 10kb up or downstream) AMT outliers assigned to the top 1% of *ZTD* in the (A) anadromous and (B) resident pools. Columns indicate chromosome name (chr), gene name, ensemble gene id, gene start and end positions and gene description.

**Table S9.A-B)** Genes associated with (within 10kb up or downstream) AMT outliers assigned to the top 1% of Betascores in the (A) anadromous and (B) resident pools. Columns indicate chromosome name (chr), gene name, ensemble gene id, gene start and end positions and gene description.

**Table S10.A-B)** Genes associated with (within 10kb up or downstream) AMT outliers assigned to the top 1% of intersex *F_ST_* in the (A) anadromous and (B) resident pools. Columns indicate chromosome name (chr), gene name, ensemble gene id, gene start and end positions and gene description.
